# Extracellular vesicles and microRNAs in metabolic dysfunction-associated steatotic liver disease: from steatosis to hepatocellular carcinoma

**DOI:** 10.17179/excli2025-8710

**Published:** 2025-10-23

**Authors:** Melina Belén Keingeski, Larisse Longo, Anelise da Silva Pinto, Bruno de Souza Basso, Thalia Michele Vier Schmitz, Vitória Brum da Silva Nunes, Juliete Nathali Scholl, Camila Kehl Dias, Fabrício Figueiró, Danieli Rosane Dallemole, Adriana Raffin Pohlmann, Isabel Veloso Pereira, Jose Tadeu Stefano, José Eduardo Vargas, Patrícia Luciana da Costa Lopez, Claudia P. Oliveira, Juan Pablo Arab, Mário Reis Álvares-da-Silva, Carolina Uribe-Cruz

**Affiliations:** 1 Graduate Program in Gastroenterology and Hepatology, Universidade Federal do Rio Grande do Sul, Porto Alegre, Rio Grande do Sul, Brazil; 2 Experimental Laboratory of Hepatology and Gastroenterology, Center for Experimental Research, Hospital de Clínicas de Porto Alegre, Porto Alegre, Rio Grande do Sul, Brazil; 3 Laboratory of Cancer Immunobiochemistry, Department of Biochemistry, Universidade Federal do Rio Grande do Sul, Porto Alegre, Rio Grande do Sul, Brazil; 4 Graduate Program in Biological Sciences: Biochemistry, Instituto de Ciências Básicas da Saúde, Universidade Federal do Rio Grande do Sul, Porto Alegre, Rio Grande do Sul, Brazil; 5 Graduate Program in Pharmaceutical Sciences, Faculty of Pharmacy, Universidade Federal do Rio Grande do Sul, Porto Alegre, Rio Grande do Sul, Brazil; 6 Laboratório de Investigação Médica (LIM07) do Hospital das Clínicas, da Faculdade de Medicina da Universidade de São Paulo, SP, Brazil; 7 Departamento de Gastroenterologia, Faculdade de Medicina da Universidade de São Paulo, São Paulo-SP, Brazil; 8 Laboratory of Inflammatory and Neoplastic Cells, Department of Cell Biology, Section of Biological Sciences, Universidade Federal do Parana, Curitiba, Brazil; 9 Conselho Nacional de Desenvolvimento Científico e Tecnológico, Brazil, CNPq researcher; 10 Division of Gastroenterology, Hepatology, and Nutrition, Department of Internal Medicine, Virginia Commonwealth University School of Medicine, Richmond, VA, 23298, USA; 11 Departamento de Gastroenterología, Escuela de Medicina, Pontificia Universidad Católica de Chile, Santiago, Chile; 12 Division of Gastroenterology, Hospital de Clínicas de Porto Alegre, Porto Alegre, Rio Grande do Sul, Brazil; 13 Centro de Investigación de la Facultad de Ciencias de la Salud, Universidad Católica de las Misiones, Posadas, 3300, Argentina

**Keywords:** cirrhosis, extracellular vesicles, hepatocellular carcinoma, metabolic dysfunction-associated steatotic liver disease, metabolic dysfunction-associated steatohepatitis, microRNAs

## Abstract

Extracellular vesicles (EVs) and microRNAs, involved in intercellular communication, have emerged as potential biomarkers in liver diseases. This study aimed to evaluate EV characteristics and microRNA transport across the full spectrum of metabolic dysfunction-associated steatotic liver disease (MASLD). 168 patients with MASLD and 50 controls were recruited. Biochemical and clinical variables were evaluated. EVs were isolated from serum and characterized by nanoparticle tracking analysis, flow cytometry, and Western blotting. Using MiRWalk 3.0 and the TarPmiR algorithm, candidate EV-associated microRNAs related to MASLD were identified. The expression of miR-4758, miR-188, miR-1226, and miR-122, was evaluated in EVs and serum. EV size and concentration varied significantly across disease stages (p<0.001 and p<0.05, respectively), with early MASLD dominated by exosome, and later stages showing a shift toward microvesicles. In MASLD patients, interestingly, miR-122 was lower in EVs compared to serum (p<0.05). In steatosis, it was higher in serum than EVs (p<0.05), without significant differences in later stages. miR-122 in EVs increased in association with GGT and cholesterol, and decreased with elevated creatinine. Serum miR-122 was also elevated in patients with high cholesterol. In MASLD miR-4758 was higher in EVs than in serum (p<0.05), expressed in steatosis and cirrhosis (p<0.05), suggesting it is a good disease marker, and detected exclusively in serum in HCC (p<0.05). miR-4758-EVs increased with high glucose. MiR-188 and miR-1226 were exclusively expressed in serum (p<0.05), and miR-1226 was elevated in patients with high cholesterol. EV size was reduced in individuals with high triglycerides and albumin, suggesting interaction between EVs, biochemical parameters and disease stage. These findings suggest that microRNA expression and transport in EVs and serum vary across MASLD stages and associate with key biochemical parameters, supporting the clinical value of jointly assessing both compartments as potential biomarkers to distinguish early disease from advanced stages such as HCC.

See also the graphical abstract(Fig. 1).

## Abbreviations

AH: arterial hypertension

ALP: alkaline phosphatase

ALT: alanine aminotransferase

AST: aspartate aminotransferase

BMI: body mass index

CHFD: choline-deficient hyperlipidic diet

EDTA: ethylenediaminetetraacetic acid

EVs:extracellular vesicles

EXOs: exosomes

GGT: gamma-glutamyl transferase

HBV: hepatitis B virus

HCV: hepatitis C virus

HDL: high-density lipoprotein

HCC: hepatocellular carcinoma

HIV: human immunodeficiency virus infection

LDL: low-density lipoprotein

MASLD: metabolic dysfunction-associated steatotic liver disease

MASH: metabolic dysfunction-associated steatohepatitis

MetS: metabolic syndrome

microRNAs: miRNAs

MVB:multivesicular bodies

MVs: microvesicles

NAFLD: non-alcoholic fatty liver disease

NTA: nanoparticle tracking analysis

PBS: phosphate-buffered saline

TBS: Tris-buffered saline

TC: total cholesterol

TTBS: Tris-buffered saline with Tween 20

WB: Western blotting

## Introduction

Metabolic dysfunction associated steatotic liver disease (MASLD), previously known as non-alcoholic fatty liver disease (NAFLD) (Rinella et al., 2023[27]). Over the last three decades, the global prevalence of MASLD has increased by 50.4 %, reflecting the growing global epidemic of obesity and type 2 diabetes mellitus (Arrese et al., 2021[2]; Younossi et al., 2023[34]). MASLD includes a range of liver conditions from steatosis to metabolic dysfunction-associated steatohepatitis (MASH), cirrhosis, and hepatocellular carcinoma (HCC) (Arrese et al., 2021[2]; Rinella et al., 2023[27]). The disease is characterized by lipotoxicity, inflammation, and fibrosis, although the mechanisms of its development are complex and not yet fully understood (Kuchay et al., 2020[16]; Loomba et al., 2021[20]).

In clinical practice, MASLD is typically diagnosed by changes in serum markers and/or visualization of lipid deposits in the liver through imaging methods. However, liver biopsy remains the gold standard for diagnosis and stratification of the disease. Therefore, to advance in the clinical management of MASLD, it is important to develop non-invasive biomarkers to improve diagnosis, risk stratification, and monitoring of disease progression (Nassir, 2022[21]; Pouwels et al., 2022[24]).

Thus, extracellular vesicles (EVs) have emerged as promising tools due to their stability in circulation and sensitivity to reflect the state of the originating cell. EVs can carry genetic information, playing a crucial role in intercellular communication and potentially serving as biomarkers in MASLD (Newman et al., 2020[23]; Srinivas et al., 2023[28]). Furthermore, microRNAs (miRNAs), which regulate gene expression in various biological and pathophysiological processes, have also become an important focus of attention as a biomarker for MASLD (Babuta and Szabo, 2022[3]). miRNAs are secreted extracellularly and transported in different forms: freely in circulation bound to Argonaute2 protein or within cells-derived EV, reflecting the state of hepatic disease (Babuta and Szabo, 2022[3]; Ge et al., 2018[11]). Previous studies conducted by our group in animal models of MASLD have shown that serum miR-122 exhibits increased expression in diseased animals compared to controls (de Freitas et al., 2022[6]; Longo et al., 2020[19]). Therefore, this study aims to explore the transport of miRNAs in humans and the role of EVs in the progression of MASLD, aiming to better characterize their potential as a biomarker.

## Materials and Methods

### Population of study

A total of 168 individuals aged ≥ 18 years of both genders were included, representing all spectrum of MASLD from isolated steatosis to HCC. The diagnosis was based on the presence of steatosis by ultrasound or liver biopsy, with or without alterations in aspartate aminotransferase (AST) and/or alanine aminotransferase (ALT), associated with metabolic syndrome (MetS). Ultrasounds performed up to six months and liver biopsies carried out up to one year before inclusion in the study were considered valid. The diagnosis of hepatocellular carcinoma secondary to MASLD was established using clinical criteria, imaging studies, or histopathological findings. The characterization of different disease stages was extracted from electronic medical records. Exclusion criteria included age < 18 years, human immunodeficiency virus infection (HIV), active hepatitis B virus (HBV) or hepatitis C virus (HCV) infection, alcohol consumption higher than 20 g or 30 g ethanol/day (women or men, respectively), pregnancy, transplant recipients, use of immunosuppressants, corticosteroids, valproic acid, tetracycline, amiodarone, and chronic inflammatory diseases. To integrate the control group, 50 individuals aged ≥ 18 years of both genders recruited in 2018 were included. These were individuals without any sign of liver disease, denying the use of medications, and not using antibiotics in the six months prior to inclusion in the study.

Whole blood samples collected from participants were processed at 3000 rpm for 10 minutes at room temperature to obtain serum, which was subsequently stored at -80°C until further use. 

### Demographic, comorbidity and biochemical characterization

In the MASLD patient, demographic variables such as age, body mass index (BMI), and sex were evaluated. Comorbidity variables assessed included active smoking, alcohol consumption, hypertension, diabetes, MetS, HBV, and HCV. Biochemical variables were analyzed: ALT, AST, gamma-glutamyl transferase (GGT), alkaline phosphatase (ALP), albumin, creatinine, total bilirubin, indirect bilirubin, triglycerides, total cholesterol (TC), high-density lipoproteins (HDL), low-density lipoproteins (LDL), glucose, leukocyte count, and platelets. Additionally, the MELD and Child-Pugh scores were calculated to assess liver function.

### Isolation of serum EV by Size-Exclusion Chromatography (SEC) and characterization Nanoparticle Tracking Analysis (NTA)

To isolate EVs, 93 serum samples from the MASLD group and 25 from the control group were processed. The procedure was based on the protocol by Théry et al. (2018[30]) with some modifications (see methodology in Supplementary information).

The particle size and size distribution curves were determined using the NTA technique on a NanoSight LM10 instrument (NanoSight Ltd., UK). This analysis is based on the light scattering of particles undergoing Brownian motion, which is captured by a charge-coupled device camera attached to a microscope operating with a 20× objective. Samples were diluted in PBS (1:5 v/v), introduced into the sample chamber using a syringe (1 ml), and analyzed using a 638 nm laser. For each sample a 60-second video was captured and subsequently processed using NTA 3.2 Dev Build 3.2.16 software. The camera level was manually adjusted for each sample to optimize particle visualization, ranging from 9-14. Meanwhile, the detection threshold was maintained at 5 for all analyses. The results were expressed as the hydrodynamic mean diameter (nm) and particle number density (particles concentration; particles/ml), with the data presented as mean and standard deviation.

### Identification of proteins in EVs by flow cytometry and Western blotting

Flow cytometry was utilized to determine the expression of CD9 and CD63 on the EVs, following the protocol outlined by Suárez et al. (2017[29]) (see methodology in Supplementary information). Additionally, the expression of GM130, Alix, and Annexin was evaluated by Western blotting (see methodology in Supplementary information).

### Prediction of microRNAs in patients

In line with a study conducted by Gim et al. in 2021 a combination of EV miRNAs extracted through microarray analysis from the serum of MASLD patients, along with analysis of variance (ANOVA), was employed to pinpoint microRNAs associated with both MASLD stages. Under each condition, potential gene targets were forecasted using MiRWalk 3.0 (http://mirwalk.umm.uni-heidelberg.de/), an easily accessible database containing predicted data derived from a machine learning algorithm (Dweep et al., 2014[8]). A maximum score, equivalent to 1, was computed through a random-forest-based approach by executing the TarPmiR algorithm for miRNA target site prediction. MicroRNAs influencing more than 200 unique genes were then chosen for verification through qPCR-real time in the context of both MASLD stages. 

After qPCR- real time, statistically significant miRNAs were utilized to design interacting networks. For this purpose, gene targets predicted by MiRWalk 3.0 were combined with the metasearch engine StringApp (score≥0.4) within the Cytoscape 3.9.1 platform (Doncheva et al., 2019[7]). Network centrality was performed to define relevant nodes in the obtained network (See the detailed description of centrality analysis and network design in the Supplementary information).

### Extraction and quantification of microRNAs in patients

From this, 3 microRNAs were chosen to influence around 200 genes and to be representative of both MASLD stages: miR-188, miR-4758, and miR-1226. Additionally, miR-122 was chosen for being a liver-specific microRNA and being involved in the disease.

The microRNAs were extracted from EV and serum using the miRNeasy serum/plasma kit (Qiagen, USA). The cel-miR-39 (1.6×10^8^ copies) spike-in control was added as an internal reference for normalization of technical variations between samples, following the manufacturer's instructions. The cDNA conversion was performed on 10 ng of total RNA using TaqMan microRNA reverse transcription kits (Applied Biosystems, USA). The gene expression analysis of miR-4758, miR-188, miR-1226 and miR-122 and its normalizer cel-miR-39 was performed using qRT-PCR with TaqMan probes (Applied Biosystems, USA) (Supplementary information, Supplementary Table 1). The values were calculated using the 2 ^-(ΔΔCt)^ formula.

### Sample size calculation and statistical analysis

The sample size calculation was based on the data presented by GENFIT at the European Association for the Study of the Liver (EASL) digital event in August 2020 (Ratziu et al., 2016[26]). To discriminate between MASLD and control participants, it was necessary to recruit more than 50 participants diagnosed with MASLD and 50 control participants. This results in a final sample size of 168 participants for the study, considering a statistical power of 90 % and a significance level of 5 %. The normality of the variables was assessed using the Shapiro-Wilk test and histograms. The statistical analysis included one-way ANOVA, two-way ANOVA with Tukey's post-hoc test, paired t-test, and Wilcoxon test. Quantitative variables were reported as mean ± standard deviation or median and interquartile range (25th-75th percentile). The level of statistical significance was set at p ≤ 0.05. The data were analyzed using the Statistical Package for Social Sciences (version 28.0; SPSS Inc., USA).

## Results

### General characteristics of the MASLD and control patients

The control group consisted of 50 healthy individuals. The mean age was 41 years (SD = 17), and the average BMI was 24.1 kg/m² (SD = 3.1). The majority were women (76 %, n = 38). None of the control participants had a diagnosis of arterial hypertension, diabetes mellitus, metabolic syndrome, or infection with hepatitis B or C virus, nor did they report active alcohol consumption or smoking.

A total of 168 participants with MASLD were included in the study: isolated steatosis (n=50), MASH (n=49), cirrhosis (n=50) and HCC (n=20). Their demographic, comorbidity, biochemical variables, and liver function scores (MELD and Child-Pugh) are summarized in Table 1(Tab. 1). When evaluating the demographic and comorbidity variables across these MASLD stages, we found a significant association (p<0.05) between hypertension, diabetes, MELD and CHILD-Pugh scores with MASLD (Supplementary Table 2a). Patients with HCC showed significantly higher MELD and Child-Pugh scores compared to those with cirrhosis (U = 307, p=0.015; U = 131.5, p=0.017, respectively). The logistic regression model revealed a significant association between liver disease stages and the presence of hypertension (p=0.049) as well as diabetes (p=0.040). In stage-specific comparisons, the model indicated that patients with steatosis were approximately 4.5 times more likely to have diabetes (OR = 4.452, 95 % CI: 1.030-19.327, p=0.030) compared to the reference group (Supplementary Table 2b). 

When evaluating the biochemical variables based on this stratification, we observed that AST, GGT, ALP, total bilirubin, indirect bilirubin, and creatinine significantly increased (p<0.001) in HCC compared to steatosis, MASH, and cirrhosis. Albumin, triglycerides, TC, white blood cell count, and platelets increased significantly (p<0.001, p<0.05) in Steatosis and MASH compared to cirrhosis and HCC, respectively (Supplementary Table 2c).

### Extracellular vesicles characterization isolated from serum in patients

**Figure 2:**
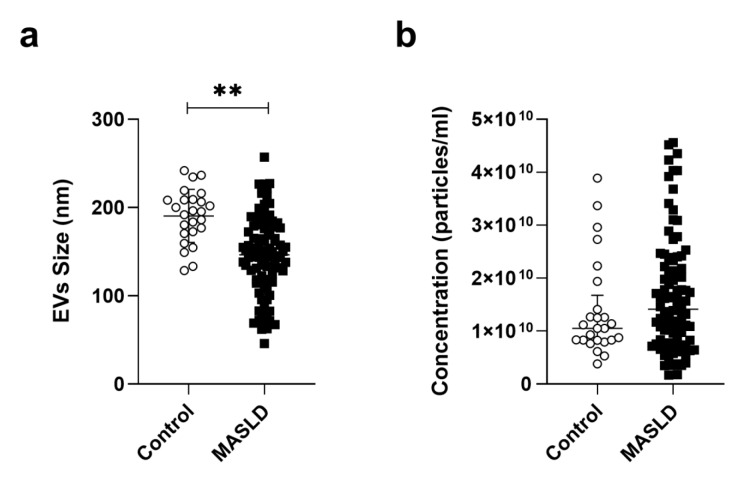
Characterization of EVs between control and MASLD patients. (a) EVs mean size. (b) EVs concentration. EVs: extracellular vesicles; MASLD: metabolic-dysfunction-associated steatotic liver disease. Data were presented as either mean ± standard deviation using the one-way ANOVA test with Tukey's post-hoc test, and median and interquartile ranges using the Mann-Whitney test. Statistical significance: *p ≤ 0.001, p ≤ 0.05.*

The EVs isolated from the MASLD group presented an average size of 146 ± 45 nm and a concentration of 1.35E^10^ (7.70E^9^ - 2.48E^10^). On the other hand, the control group showed an average size of 190 ± 30 nm and a concentration of 1.05E^10^ (1.01E^10^ - 1.78E^10^) (Figure 2a-b(Fig. 2)). These data indicated a significant decrease in the size of EVs in the MASLD group (p< 0.001) compared to the control group. 

**Figure 3:**
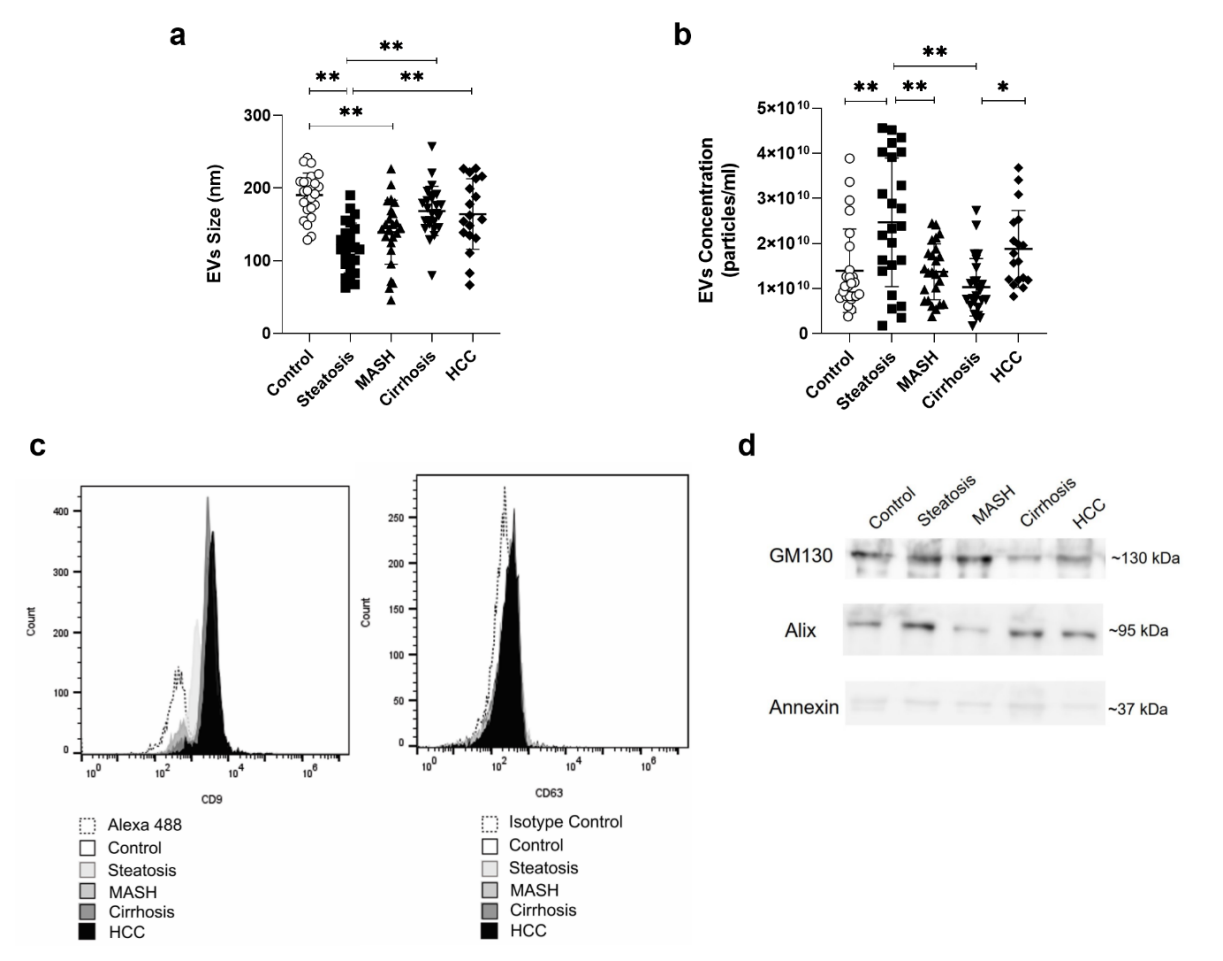
Characterization of EVs isolated from patients' serum. (a) EVs mean size (nm). (b) EVs concentration (particles/ml). (c) Representative image showing CD9 and CD63 protein expression in EV samples per group. (d) Protein identification by western blot, with a representative image for each group. EVs: extracellular vesicles; HCC: hepatocellular carcinoma; MASH: metabolic dysfunction-associated steatohepatitis. Data are presented as mean ± standard deviation using the one-way ANOVA test with Tukey's post-hoc test. Statistical significance: *p ≤ 0.001, p ≤ 0.05.*

When evaluating the mean size of EVs in the different stages of MASLD according to the progression, the Steatosis and MASH stage indicated the presence of exosomes (<150 nm), while in the control, cirrhosis, and HCC stage were microvesicles (150-1000 nm) (Figure S1). The mean size of the vesicles was significantly dependent on the disease stage (p<0.001). The HCC group (164±49 nm) increased significantly compared to the Steatosis group (117±34 nm). The cirrhosis group (168±34 nm) showed a significant increase compared to the Steatosis group. The MASH group (139±44 nm) and Steatosis decreased significantly compared to the control group (190±30 nm) (Figure 3a(Fig. 3)).

The concentration of EVs also varied with the disease stages. There was a significant increase in the HCC group (1.88±0.85) x10^10^ particles/mL^-1^ compared to the cirrhosis group (1.03±0.64) ×10^10^ particles/mL^-1^ [p< 0.05]. The cirrhosis group exhibited a significant decrease compared to the steatosis group (2.47±1.43) ×10^10^ particles/mL^-1^. [p<0.001]. The MASH group (1.37±0.62) ×10^10^ particles/mL^-1^ showed a significant decrease compared to the steatosis group (p<0.001). The steatosis group increased significantly compared to the control group (1.39±0.93) ×10^10^ particles/mL^-1^ [p<0.05] (Figure 3b(Fig. 3)).

On the other hand, EVs isolated from the serum of the MASLD and control groups are positively stained for the membrane tetraspanins CD9 and CD63 (Figure 3c(Fig. 3)) and indicated presence of the proteins Alix, GM130, and Annexin (Figure 3d(Fig. 3); Figure S2-S4).

### Expression of microRNAs isolated from extracellular vesicles and serum in patients

In a small preliminary experiment in an animal model of MASLD (see methodology in Supplementary information), we found that miR-122 was expressed within EV without significant difference between diseased and control groups. Nonetheless, we observed an increase in the expression of miR-122 in serum in the diseased groups, especially in those in advanced stages. Given this difference, we proceeded to compare the expression of miR-122 between these two sample types only in the diseased groups, observing that in advanced stages, miR-122 was highly expressed in serum when compared to EV (Figure S5). Due to these findings, we analyzed the expression of serum microRNAs and in EV in human samples.

**Figure 4:**
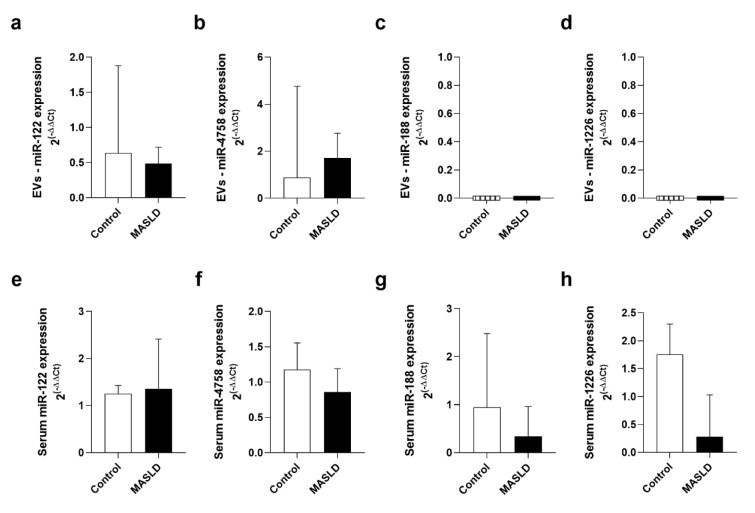
Expression of miRNAs from EVs and serum in control and MASLD. (a) miR-122 expression in EVs. (b) miR-4758 expression in EVs. (c) miR-188 expression in EVs. (d) miR-1226 expression in EVs. (e) miR-122 expression in serum. (f) miR-4758 expression in serum. (g) miR-188 expression in serum. (h) miR-1226 expression in serum. EVs: extracellular vesicles; MASLD: metabolic-dysfunction-associated steatotic liver disease. Data were presented as median and interquartile range using the Mann-Whitney test. Statistical significance: *p ≤ 0.001, p ≤ 0.05.*

When evaluating the expression of miR-122, miR-4758, miR-188, and miR-1226, the results showed no difference in gene expression between MASLD groups and controls within vesicles (Figure 4a-d(Fig. 4)) and in serum (Figure 4e-h(Fig. 4)). It is noteworthy that miR-188 and miR-1226 were expressed only in serum (Figure 4g-h(Fig. 4)).

**Figure 5:**
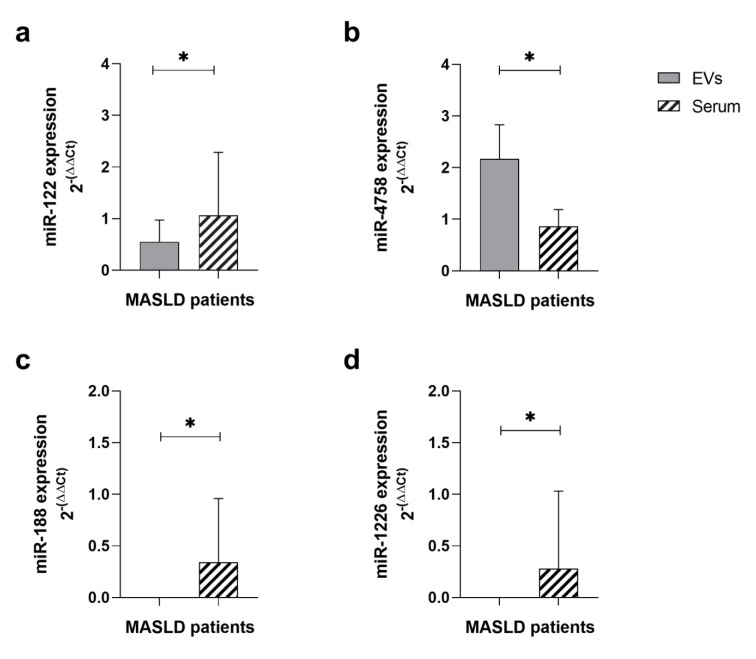
miRNA gene expression in EVs and serum in MASLD patients. (a) miR-122. (b) miR-4758. (c) miR-188. (d) miR-1226. EVs: extracellular vesicles; MASLD: metabolic-dysfunction-associated steatotic liver disease. Data shown as median and interquartile ranges. Wilcoxon test. Statistical significance: *p ≤ 0.001, p ≤ 0.05.*

When observing that certain microRNAs associated with disease development, such as miR-188 and miR-1226, were absent within the EV but present in the serum, we compared their expression, in MASLD patients, between EVs and free serum. Thus, we observed that miR-122 had a significantly lower expression within the EV compared to serum (p<0.05). miR-4758 showed a significant increase in gene expression within the EV compared to serum (p<0.05). On the other hand, miR-188 and miR-1226 were found to be expressed exclusively in the serum (p<0.05) (Figure 5a-d(Fig. 5)).

**Figure 6:**
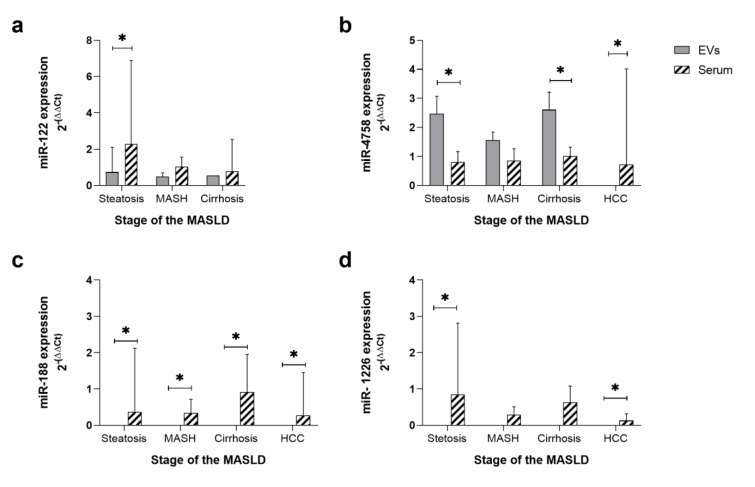
Comparison of microRNA expression between EVs and serum at each stage of MASLD. (a) miR-122. (b) miR-4758. (c) miR-188. (d) miR-1226. EVs: extracellular vesicles; HCC: hepatocellular carcinoma; MASH: metabolic dysfunction-associated steatohepatitis; MASLD: metabolic-dysfunction-associated steatotic liver disease. Data shown as median and interquartile ranges. Wilcoxon test. Statistical significance: *p ≤ 0.001, p ≤ 0.05.*

After observing the difference in expression between EVs and serum, we attempted to find an explanation following the disease progression. microRNAs exhibit different expressions depending on the stage of the disease being evaluated. miR-122 showed higher expression in serum compared to its expression within EV in the Steatosis group (p<0.05); however, in later stages, there was no difference. It is important to note that it was not possible to evaluate the expression of miR-122 in HCC patients due to technical issues (Figure 6a(Fig. 6)). MiR-4758 was significantly expressed within EV in the steatosis and cirrhosis groups (p<0.05). However, in HCC patients, it was solely expressed in serum (Figure 6b(Fig. 6)). As mentioned earlier, miR-188 and miR-1226 were solely expressed in the serum of MASLD (p<0.05) (Figure 6c-d(Fig. 6)).

### Correlation analysis between extracellular vesicles, microRNAs, and biochemical/clinical variables in MASLD 

In the overall MASLD group, the size of EVs showed a moderate negative correlation with ALT (p<0.05), and strong negative correlations with albumin and TG levels (p<0.01). miR-4758-EV exhibited moderate positive correlations with total bilirubin and glucose (p<0.05). miR-122-EV demonstrated a strong negative correlation with creatinine levels (p<0.05) (Supplementary Table 3). 

When analyzing the associations by subgroup, in the steatosis group, the size of EV showed moderate to strong negative correlations with GGT and total cholesterol (p<0.05), and a moderate negative correlation with ALT (p<0.05). miR-4758-EV showed a strong positive correlation with total bilirubin (p<0.05), while miR-188 in serum was moderately and positively correlated with HDL (p<0.05) (Supplementary Table 3a). In the MASH group, miR-122 in serum showed strong negative correlations with ALP and albumin (p<0.05) (Supplementary Table 3b). In the cirrhosis group, the size of EVs showed a moderate positive and negative correlation with HDL (p<0.05) and glucose (p<0.01), respectively. Additionally, both miR-4758-EV and miR-4758 in serum exhibited moderate negative correlations with glucose (p<0.05) (Supplementary Table 3c). In the HCC group, exploratory analysis showed very strong positive correlations between miR-122 in serum and both total cholesterol and LDL (p<0.05). These findings should be interpreted with caution due to the limited sample size (n=5) (Supplementary Table 3d).

### Differential EVs and miRNA profiles by high vs. low biochemical marker levels

To further explore associations with clinical parameters, we analyzed EVs and miRNA characteristics in relation to biochemical markers.

Higher levels of miR-122-EV were observed in patients with elevated GGT while lower levels of miR-122-EV were found in those with high creatinine (p<0.05, respectively). In addition, miR-122 and miR-1226 in serum were higher in patients with elevated total cholesterol (p<0.05). miR-4758-EV levels were higher in patients with elevated glucose (p=0.050). Smaller EV size was associated with both high triglyceride levels (p<0.01) and high albumin levels (p<0.01) (Figure S6). No significant associations were found between EVs or miRNAs and ALT, AST, total bilirubin, HDL, LDL, or alkaline phosphatase.

## Discussion

Currently, microRNAs and extracellular vesicles (EVs) are being explored as potential biomarkers for metabolic-dysfunction-associated steatotic liver disease (MASLD). However, the mechanisms underlying their expression and transport across different disease stages are not fully understood. In this study, we demonstrated that EV size and concentration vary according to MASLD stages. Moreover, miRNAs in serum and EVs could provide significant information related to disease severity. Additionally, we investigated the association of these molecular parameters with biochemical markers and liver function scores, aiming to understand their potential role in reflecting disease progression.

In a previous study, we evaluated the concentration and size of EVs in an experimental MASLD model induced by a choline-deficient hyperlipidic diet (CHFD). Additionally, we assessed how the EVs correlate with inflammatory markers of disease progression (Keingeski et al., 2024[15]). Our results indicated that EVs are associated with different types of inflammatory cytokines depending on the stage of the disease, pro-inflammatory cytokines in early stages and anti-inflammatory in advanced stages. In other words, EVs carry molecules that reflect distinct processes in MASLD (Keingeski et al., 2024[15]). Therefore, with this model, we set out to investigate whether these EVs also contain microRNAs. Our data demonstrated that miR-122 expression increased as the disease progressed, as already reported in the literature (Jiang et al., 2021[14]; Povero et al., 2014[25]). When comparing the type of transport in diseased animals, we observed that miR-122 primarily circulates freely in the serum at advanced stages, with no difference observed in early stage. This raises questions about how microRNAs are expressed and transported in MASLD. For this purpose, we conducted this clinical study in patients with different stages of MASLD. 

As expected, the biochemical alterations changed as the disease progressed. Additionally, our data highlight the importance of considering liver disease progression when assessing the risk of both hypertension and diabetes. Steatosis was found to potentially play a significant role in the predisposition to diabetes, while other stages of MASLD showed no association with hypertension or diabetes, consistent with previous findings (Hsu and Loomba, 2024[13]). The associations identified through logistic regression reinforce the clinical relevance of these findings in managing hepatic and metabolic health. Furthermore, although fewer patients had HCC, their significantly higher Child-Pugh and MELD scores indicate more advanced hepatic dysfunction compared to those with cirrhosis This finding supports the notion that the development of HCC in MASLD may be associated with greater hepatic decompensation. These results align with previous evidence recognizing both Child-Pugh and MELD scores as reliable indicators of liver functional reserve and as key components in HCC staging and prognosis (Tsoris and Marlar, 2023[31]; Zhao et al., 2024[35]; Zhao et al., 2020[36]).

When characterizing EVs in samples from MASLD patients, our results revealed that in the early stages of the disease (steatosis and MASH) the predominant population of EVs was exosomes (EXOs; 30-150 nm). Conversely, in advanced stages (cirrhosis and HCC), the predominant population observed was microvesicles (MVs; <1000 nm). In the control group, we also found MVs. These findings suggest that, as the disease progresses, cells release different populations of EVs, reflecting distinct mechanisms of intercellular communication. The biogenesis of EXOs begins with the formation of vesicles in early endosomes of cells, which then develop into multivesicular bodies (MVBs), fuse with the plasma membrane, and release their contents into the extracellular environment (Newman et al., 2022[22]; Xu et al., 2022[33]). In contrast, MVs originate directly from the plasma membrane through budding or budding-off processes, leading to the formation and release of MVs into the extracellular milieu (Newman et al., 2022[22]; Xu et al., 2022[33]). 

Additionally, in these patients, we assessed the expression of four microRNAs (miR-122, miR-4758, miR-188, and miR-1226) that target genes involved in MASLD progression (Figure S7), such as *HMGCR,* *ADORA2A, IL-15, VCAM1, CXCR2, FBXW7 e NOTCH4.* MiR-122 is the most well-known and prevalent microRNA in hepatic tissue, considered a key positive regulator of de novo lipogenesis and essential for hepatocyte differentiation and development (Fang et al., 2021[10]). On the other hand, our predictive analysis indicated that miR-4758, miR-188, and miR-1226 are involved in regulating the expression of genes implicated in lipid accumulation, inflammatory response, immune cell recruitment, hepatic injury, fibrosis progression, migration, and invasion of hepatic tumor cells. These findings are summarized in Supplementary Table 4.

The four microRNAs did not show differences between MASLD patients and controls. However, within the MASLD group, we found that miR-122 and miR-4758 were expressed both in serum and in EVs, whereas miR-188 and miR-1226 were present only in serum. These findings are consistent with results previously discovered in animal models. To assess whether differences in transport were associated with disease progression, we compared the expression of miRNAs in EV and serum across different disease stages. Overall, we observed a shift in the transport pattern of miRNAs throughout MASLD. Specifically, miR-122 showed high expression in serum during the early stages. However, as the disease advanced, no significant difference between EV and serum was detected. MiR-4758 exhibited high expression in EV during steatosis and cirrhosis, but in HCC, was exclusively transported in serum. As previously mentioned, miR-188 and miR-1226 were consistently expressed only in serum across all MASLD.

These data suggest that microRNAs transported within EV and those circulating freely have distinct biological roles and implications. MicroRNAs contained in EV are recognized as key regulators of pathological processes and are considered more stable in peripheral circulation. In contrast, freely circulating microRNAs might be more susceptible to degradation and could have different regulatory functions. One study demonstrated that microRNAs in EVs are less abundant compared to freely circulating microRNAs when measured by blood plasma volume. Furthermore, the increase in circulating microRNAs may be explained by their stability when bound to Argonaute2 protein (Turchinovich et al., 2011[32]), which aligns with our findings of increased serum microRNAs. Additionally, Turchinovich et al. 2011 proposed that microRNA/Argonaute2 complexes are primarily derived from apoptotic or necrotic cells, which might explain why our miR-4758 was exclusively expressed in serum during the HCC stage (Turchinovich et al., 2011[32]).

Beyond the stage-related patterns, an exploratory analysis of biochemical subgroups provided additional functional insight. miR-122-EV increase in patients with high GGT, consistent with previous studies reporting simultaneous elevations of GGT and vesicular miR-122 under cholestatic conditions (Fagoonee et al., 2025[9]). In contrast, elevated creatinine-an indicator of impaired glomerular filtration-was accompanied by lower miR-122-EV, suggesting loss of the tubular export of this anti-inflammatory microRNA and, consequently, unchecked renal inflammation (Li et al., 2024[18]). Additionally, serum miR-122 was higher in participants with elevated total cholesterol, in line with clinical studies linking circulating miR-122 to adverse lipid profiles (Abdelhafez et al., 2024[1]). By contrast, evidence connecting miR-1226 with dyslipidemia remains limited. Tumour‐based studies show that miR-1226 can drive M2 macrophage polarization and promote cell survival (Choi et al., 2024[5]) but its metabolic role is still unclear. The overexpression observed in patients with high total cholesterol may therefore reflect a broader state of metabolic stress rather than a direct lipid-regulatory function. 

Currently, no direct evidence links hyperglycemia to an increase in miR-4758-EV. One study reported lower miR-4758 levels in peripheral blood mononuclear cells after a prolonged ketogenic (low-carbohydrate) diet; however, that work did not analyze EV and was conducted under hypoglycemic conditions (Chodur and Steinberg, 2024[4]). Another study mentioned several glucose-responsive microRNAs (e.g., miR-223, miR-192-5p) but did not include miR-4758 (Lee et al., 2025[17]). Taken together, our observation of higher miR-4758-EV in patients with elevated glucose appears biologically plausible-hyperglycemia is known to reprogram EV cargo-yet targeted studies combining glucose challenge with miR-4758 quantification in EV are needed to confirm or refute this association.

Finally, in patients with elevated triglycerides and albumin, we observed EV of smaller size, consistent with studies showing that lipid overload and preserved hepatic synthetic function favor the release of small, exosome-like particles (Fagoonee et al., 2025[9]; Zhao et al., 2020[37]). 

The study has some limitations: it lacked liver-specific EV markers, did not assess pro-inflammatory or anti-inflammatory cytokines in the EV, and could not analyze miR-122 in HCC due to an insufficient sample size of patients with advanced disease. Additionally, we did not explore the specific gene target of the microRNAs. These factors should be considered when interpreting the findings, and further research is recommended.

Therefore, we suggest that it is important for future studies to evaluate microRNA expression both within EV and in serum. This approach may help clarify their roles and further supports the growing notion of using microRNAs and EV as biomarkers in MASLD.

## Conclusion

Our results demonstrate several key points: 1) This is the first study to assess EV across all stages of MASLD, providing a comprehensive view of disease progression. 2) Different EV populations exist according to the early or advanced stages of analyzed MASLD, allowing for their distinction. 3) We evaluated the expression and transport of microRNAs in patients with MASLD, addressing the still incomplete understanding of their expression and mode of transport during disease progression. 

MicroRNAs are considered potential biomarkers for MASLD. Although we did not observe differences in expression between MASLD patients and controls, we identified a shift in their transport pattern at advanced disease stages. This highlights the importance of jointly evaluating both microRNAs within EV and in serum, offering a more comprehensive perspective on their role as biomarkers and contributing to the understanding of MASLD pathophysiology. Moreover, correlations between EV features and biochemical markers, including liver function scores, reinforce their clinical relevance as indicators of disease progression. These findings open new avenues for future research and clinical applications.

## Notes

Mário Reis Álvares-da-Silva and Carolina Uribe-Cruz (Experimental Laboratory of Hepatology and Gastroenterology, Center for Experimental Research, Hospital de Clínicas de Porto Alegre, Rua Ramiro Barcelos, n° 2350, 2° andar Santa Cecília, Porto Alegre 90035-903, Rio Grande do Sul, Brazil; E-mail: carolinaurib10@yahoo.com.ar) contributed equally as corresponding author.

## Declaration

### Ethics approval and consent to participate

All experiments and procedures, including the use of experimental animal samples, were approved by the Institutional Ethics Committee (Protocol number: 2020-0279). All procedures were conducted in accordance with the Guide for the Care and Use of Laboratory Animals (8th ed. 2011) and Law number 11,794 (Brazil, 2008).

The collection of data and biological samples from the MASLD and control groups were approved by the Ethics Committee of Hospital de Clínicas de Porto Alegre (CAAE: 38556920.2.0000.5327 and CAAE: 94281118.7.0000.5327 respectively). 

Informed consent was obtained from all individual participants included in the study. 

### Consent for publication

Not applicable.

### Availability of data and materials

All data generated or analyzed during this study are included in this published article and its supplementary information file as well as its supplementary data file, the latter contains anonymized individual-level data.

### Funding

This study was supported by the following Brazilian funding agencies: Financiamento e Incentivo à Pesquisa from Hospital de Clínicas de Porto Alegre (FIPE/HCPA) (Dr. M.R. Álvares-da-Silva, grant 2020-0279, 2020-0516 and 2022-0078); National Council for Scientific and Technological Development - CNPq (Dr. M.R. Alvares-da-Silva, grant 423296/2021-3); Coordination for the Improvement of Higher Education Personnel - CAPES/PNPD, Brazil; the Chilean government through the Fondo Nacional de Desarrollo Científico y Tecnológico - FONDECYT (1200227 to JPA).

### Acknowledgments

Not applicable.

### Conflict of interest

The authors declare that they have no competing interest.

### Artificial Intelligence (AI) - Assisted Technology

Artificial intelligence tools were used solely for English language correction.

### Authors' contribution

Keingeski MB conducted the investigation, methodology, formal analysis, data curation, and original draft writing. Longo L contributed to the methodology, reviewed and edited the manuscript, and visualization. Pinto SA and Schmitz TMV contributed to produce and maintain research data to data curation, Souza B and Vargas J contributed to methodology and formal analysis. Nunes V, Dallemolle D, Pohlmann AR, Scholl JN, Dias KC, Figueiro F, and Pereira IV contributed to the methodology, resources, and data curation. Lopez PC was responsible for terms and conceptualization. Stefano JT contributed research data for data curation. Arab JP and Oliveira CP contributed to the methodology and conceptualization. Álvares-da-Silva MR and Uribe-Cruz C were responsible for investigation, conceptualization, methodology, funding acquisition and writing- review, and editing. All authors reviewed and approved the final manuscript.

## Supplementary Material

Supplementary information

Supplementary data

## Figures and Tables

**Table 1:**
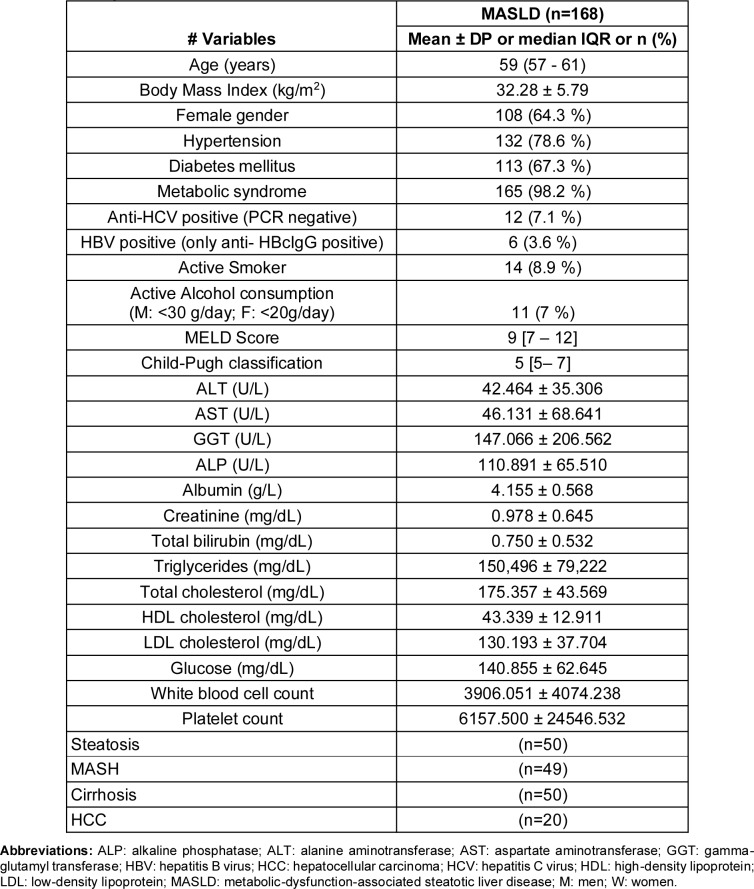
Demographic, clinical, biochemical characteristics, and liver function scores of MASLD patients

**Figure 1:**
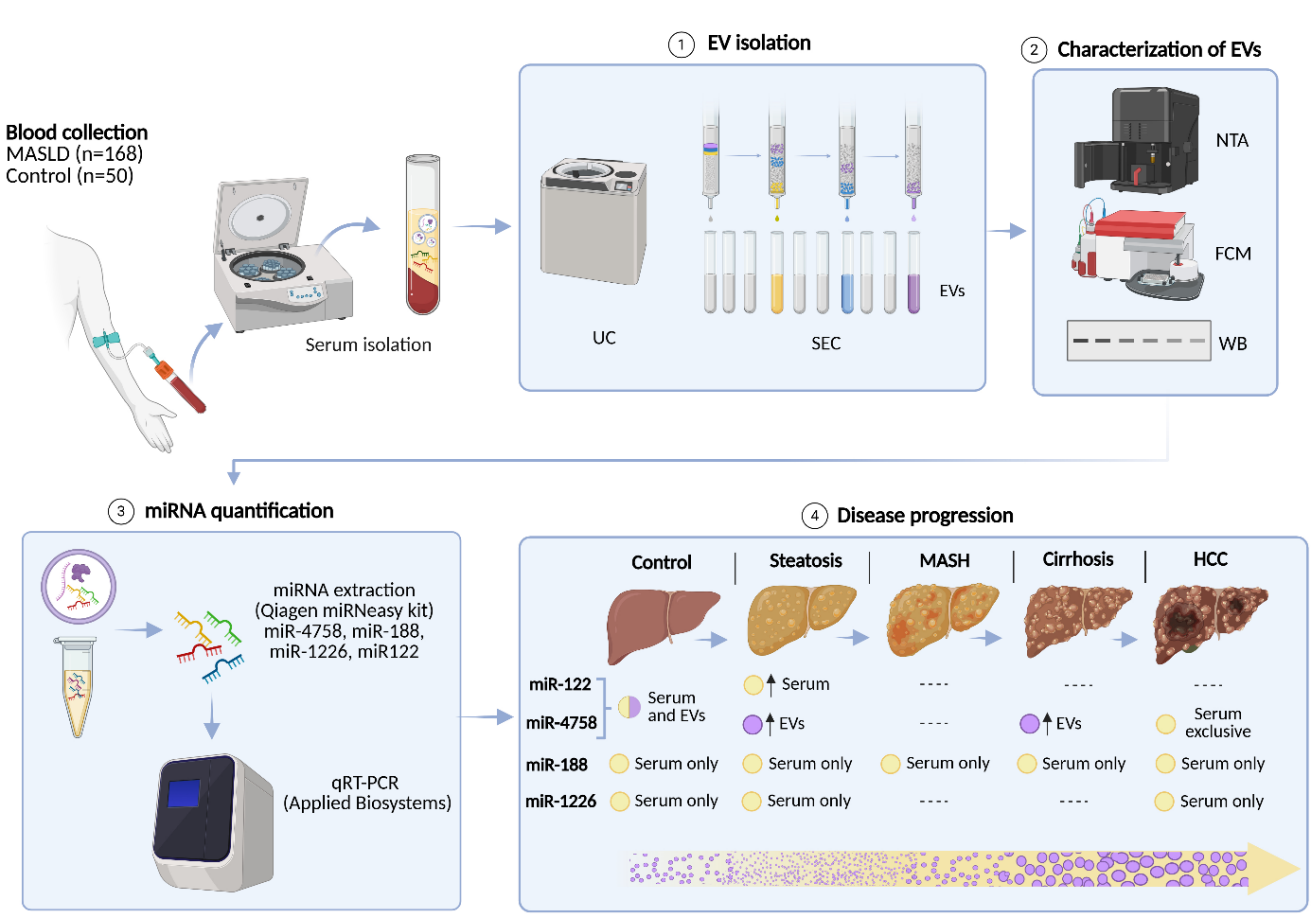
Graphical abstract: Extracellular vesicle characterization and microRNA profiles across MASLD stages. Serum samples from patients with MASLD (n = 168) and controls (n = 50) were processed to isolate extracellular vesicles (EVs) by ultracentrifugation (UC) followed by size-exclusion chromatography (SEC). EVs were characterized by flow cytometry (FCM), nanoparticle tracking analysis (NTA), and Western blot (WB). miRNAs were extracted from both EVs and serum and quantified by qRT-PCR. miR-122 was predominantly detected in serum, whereas miR-4758 was enriched in EVs at earlier stages and became serum-exclusive in HCC. miR-188 and miR-1226 were detected only in serum across all stages. Yellow circles: serum-derived miRNAs; lilac circles: EV-associated miRNAs; bottom arrow: variation in EV concentration and size throughout disease progression in serum; exosomes: small light-lilac circles; microvesicles: larger dark-lilac circles.
