# Supplementary material for: Extracellular vesicles and microRNAs in metabolic dysfunction-associated steatotic liver disease: from steatosis to hepatocellular carcinoma
Source: EXCLI J. 2025 Oct 23;24:1438–55. doi: 10.17179/excli2025-8710 (PMC12598109; doi:10.17179/excli2025-8710)
Supplement: Supplementary information [file EXCLI-24-1438-s-001.pdf]

EXCLI Journal 2025;24:1438-1455 – ISSN 1611-2156
Received: July 02, 2025, accepted: October 07, 2025, published: October 23, 2025

Supplementary information to:

Original article:

EXTRACELLULAR VESICLES AND MICRORNAS IN METABOLIC DYSFUNCTION-ASSOCIATED STEATOTIC LIVER DISEASE: FROM STEATOSIS TO HEPATOCELLULAR CARCINOMA

Melina Belén Keingeski[1,2], Larisse Longo[1,2], Anelise da Silva Pinto[1,2], Bruno de Souza Basso[1,2], Thalia Michele Vier Schmitz[2], Vitória Brum da Silva Nunes[3,4], Juliete Nathali Scholl[3,4], Camila Kehl Dias[3,4], Fabrício Figueiró[3,4], Danieli Rosane Dallemole[5], Adriana Raffin Pohlmann[5], Isabel Veloso Pereira[8,9], Jose Tadeu Stefano[8,9], José Eduardo Vargas[6], Patrícia Luciana da Costa Lopez[1], Claudia P. Oliveira[8,9,10], Juan Pablo Arab[11,12], Mário Reis Álvares-da-Silva[1,2,7,10*], Carolina Uribe-Cruz[1,2,13*]

1 Graduate Program in Gastroenterology and Hepatology, Universidade Federal do Rio Grande do Sul, Porto Alegre, Rio Grande do Sul, Brazil
2 Experimental Laboratory of Hepatology and Gastroenterology, Center for Experimental Research, Hospital de Clínicas de Porto Alegre, Porto Alegre, Rio Grande do Sul, Brazil
3 Laboratory of Cancer Immunobiochemistry, Department of Biochemistry, Universidade Federal do Rio Grande do Sul, Porto Alegre, Rio Grande do Sul, Brazil
4 Graduate Program in Biological Sciences: Biochemistry, Instituto de Ciências Básicas da Saúde, Universidade Federal do Rio Grande do Sul, Porto Alegre, Rio Grande do Sul, Brazil
5 Graduate Program in Pharmaceutical Sciences, Faculty of Pharmacy, Universidade Federal do Rio Grande do Sul, Porto Alegre, Rio Grande do Sul, Brazil
6 Laboratory of Inflammatory and Neoplastic Cells, Department of Cell Biology, Section of Biological Sciences, Universidade Federal do Parana, Curitiba, Brazil
7 Division of Gastroenterology, Hospital de Clínicas de Porto Alegre, Porto Alegre, Rio Grande do Sul, Brazil
8 Laboratório de Investigação Médica (LIM07) do Hospital das Clínicas, da Faculdade de Medicina da Universidade de São Paulo, SP, Brazil
9 Departamento de Gastroenterologia, Faculdade de Medicina da Universidade de São Paulo, São Paulo-SP, Brazil
10 Conselho Nacional de Desenvolvimento Científico e Tecnológico, Brazil, CNPq researcher
11 Division of Gastroenterology, Hepatology, and Nutrition, Department of Internal Medicine, Virginia Commonwealth University School of Medicine, Richmond, VA, 23298, USA
12 Departamento de Gastroenterología, Escuela de Medicina, Pontificia Universidad Católica de Chile, Santiago, Chile
13 Centro de Investigación de la Facultad de Ciencias de la Salud, Universidad Católica de las Misiones, Posadas, 3300, Argentina

EXCLI Journal 2025;24:1438-1455 – ISSN 1611-2156
Received: July 02, 2025, accepted: October 07, 2025, published: October 23, 2025

* **Corresponding authors:** Carolina Uribe-Cruz, Experimental Laboratory of Hepatology and Gastroenterology, Center for Experimental Research, Hospital de Clínicas de Porto Alegre, Rua Ramiro Barcelos, n° 2350, 2° andar Santa Cecília, Porto Alegre 90035-903, Rio Grande do Sul, Brazil, E-mail: carolinaurib10@yahoo.com.ar
Mario Reis Alvares-da-Silva, Division of Gastroenterology, Hospital de Clínicas de Porto Alegre, Rua Ramiro Barcelos, n° 2350/ sala 2033, 2° andar Santa Cecília, Porto Alegre 90035-903, Rio Grande do Sul, Brazil, E-mail: marioreis@live.com

https://dx.doi.org/10.17179/excli2025-8710

This is an Open Access article distributed under the terms of the Creative Commons Attribution License (https://creativecommons.org/licenses/by/4.0/).

Content

Supplementary Table 1: microRNA probes used in RT-qPCR
Supplementary Table 2a: Demographic variables, comorbidities, and liver function scores across MASLD stages
Supplementary Table 2b: Logistic regression analysis of comorbidities associated with different stages of MASLD
Supplementary Table 2c: Biochemical variables assessed at different stages of MASLD
Figure S1: Size distribution curves representative of vesicular populations in each patient group
Figure S2: GM130 Protein Expression (~130 kDa)
Figure S3: Alix Protein Expression (~95 kDa).
Figure S4: Annexin Protein Expression (~35 kDa)
Figure S5: Expression of miR-122 in experimental MASLD models
Supplementary Table 3: Correlation Analysis Between Extracellular Vesicles, microRNAs, and Biochemical/Clinical Variables in MASLD
Supplementary Table 3a: Correlation between EVs, microRNAs, and biochemical variables in Steatosis group
Supplementary Table 3b: Correlation between EVs, microRNAs, and biochemical variables in MASH group
Supplementary Table 3c: Correlation between EVs, microRNAs, and biochemical variables in cirrhosis group
Supplementary Table 3d: Correlation between EVs, microRNAs, and biochemical variables in HCC group
Figure S6: Differential EV and miRNA profiles by high vs. low biochemical marker levels
Figure S7: Systems biology analysis identifying highly connected predicted target genes
Supplementary Table 4: microRNA targets and their associated genes in MASLD
Supplementary Table 5: Raw individual-level data (see supplementary data file)

EXCLI Journal 2025;24:1438-1455 – ISSN 1611-2156
Received: July 02, 2025, accepted: October 07, 2025, published: October 23, 2025

SUPPLEMENTARY INFORMATION

MATERIALS AND METHODS

Serum Extracellular Vesicles (EVs) isolation using size-exclusion chromatography (SEC)

Serum 1ml was centrifuged at 2000 xg for 10 min, and then 10,000 xg for 30 min at 4°C. Clarified serum was further filtered using a 0.22 µm pore filter to remove large microvesicles and large lipoproteins, and it was used for subsequent EVs isolation. SEC-based isolation was conducted following previous reports (Théry et al., 2018). In short, Sepharose 2B (Sigma-Aldrich, St. Louis, Missouri, USA) was packed into 1.5 cm x 12 cm mini-columns (Bio-Rad, Hercules, Econo-Pac columns, California, USA;) with a column bed volume of 20 ml. After column washing with phosphate-buffered saline (PBS), 1ml of clarified serum was loaded onto the column, and the eluate was considered as fraction #0. Subsequently, 1 ml of PBS was repeatedly added, and fraction #4 was collected for a downstream analysis because this major fraction contained unclustered morphologically intact EVs.

Identification of proteins in EVs by flow cytometry and Western blotting

Latex beads (4 µm) capable of binding to EVs were employed (ThermoFisher, USA). Initially, bead-coupled EVs were incubated with the primary Antibody CD9 (1:200, clone: M-L13, BD Biosciences, USA) for 30 min at room temperature. Subsequently, they were washed with a blocking buffer (PBS + 2 % SFB- of bovine fetal serum), and stained with goat anti-mouse Alexa Fluor™ 488 (1:100, ThermoFisher, USA) for 30 min at 4°C. After incubation, samples were washed twice with a blocking buffer, followed by staining with anti-CD63-PE (Phycoerythrin) (1:30, clone: H5C6, BD Biosciences, USA) for 30 min at 4°C. Later, bead-coupled EVs were washed twice and analyzed using the BD Accuri C6 flow cytometer and FlowJo software (BD Biosciences, USA).

For western blotting analysis, EVs samples were homogenized in a solution containing Triton 10x, β-mercaptoethanol, Tris-buffered Saline (TBS), Ethylenediaminetetraacetic Acid (EDTA), and proteases inhibitor cocktail (Ultra Cruz protease Inhibitor, sc29131, USA). Samples were normalized to 40µg of protein. Proteins were separated with electrophoresis (polyacrylamide gel 12 % w/v) and transferred to a nitrocellulose membrane. The blot was washed with Tris-buffered Saline with Tween 20 (TTBS), followed by a 1h incubation in a blocking solution containing 3 % BSA in TTBS. After blocking, the blot was washed 3 times with TTBS and incubated overnight at 4°C with primary antibodies: anti- Actin (Sigma-Aldrich, USA) e anti-Alix, anti-GM130, anti-Annexin of kit Exosomal (Cell Signaling, USA). The primary antibodies were diluted to a concentration of 1:1000. Following overnight incubation, the blot was washed 3 times with TTBS and incubated for 2h with a horseradish peroxidase-conjugated anti-IgG secondary antibody in TBBS in a concentration of 1:2000. Clarity Western ECL Substrate (BioRad) was used for band detection, and the resulting image was captured using a ImageQuant LAS 500 (GE Life Sciences). Duplicates were performed for each group except for the HCC group. A separate membrane was prepared for each antibody.

Network design and centrality analysis

For centrality analysis, degree, betweenness, and eigenvector parameters were computed for each microRNA network using the Cytoscape platform, CentiScaPe 2.2 (Jordan et al., 2015). Centrality degree represents the count of neighboring nodes connected to a specific node. In this study, the average centrality degree was calculated as the sum of node degree scores divided by the total number of connections in the examined network. Another centrality parameter, betweenness, was investigated, representing the number of shortest paths between two nodes that pass through a specific node. Similar to the average degree parameter, the average

EXCLI Journal 2025;24:1438-1455 – ISSN 1611-2156
Received: July 02, 2025, accepted: October 07, 2025, published: October 23, 2025

betweenness was computed. Finally, eigenvector analysis was employed to assess a node's regulatory potential based on the relevance of its neighbors.

Nodes with above-average scores in node degree analysis were labeled as Hub (H), those with above-average scores in betweenness analysis were identified as Bottleneck (B), and nodes with above-average scores in eigenvector analysis were designated as Switch (S). Nodes categorized as H, B, and S collectively represent robust networking (Scardoni et al., 2014; Scardoni and Lau, 2012). Venn diagrams were generated using an online Venn tool (http://bioinformatics.psb.ugent.be/webtools/Venn/).

Extraction and quantification of microRNAs in patients

Supplementary Table 1: microRNA probes used in RT-qPCR

Assay Name	miRBAse Accession Number	Assay ID
cel-miR-39-3p	MIMAT0000010	000200
hsa-miR-4758-3p	(MIMAT0019904)	464865_mat
hsa-miR-188-5p	(MIMAT0000457)	002320
hsa-miR-1226-3p	MIMAT0005577)	245467_mat
hsa-miR-122-5p	(MIMAT0000421)	002245_mir

Experimental models of metabolic dysfunction associated steatotic liver disease (MASLD)

To obtain an indication of miR-122 transport, we utilized miR-122 serum expression data from two animal models that mimic the early and advanced stages of MASLD, previously published by our group (de Freitas et al., 2022; Longo et al., 2020). Animal models of MASLD were induced with a choline-deficient hyperlipidic diet (CHFD), for 16 and 28 weeks (MASLD-16 and MASLD-28 group respectively). The animals exhibited increased body weight, altered serum levels of aminotransferases, and hepatic changes. Through these models, we obtained a representation of both the early and advanced stages of the disease, allowing us to investigate new non-invasive methodologies (de Freitas et al., 2022; Longo et al., 2020). Additionally, we used serum-derived extracellular vesicles (EVs) from these models obtained in a previous study by Melina K. et al. 2023 (Keingeski et al., 2024) In those serum EVs, we quantified the expression of miR-122.

QUANTIFICATION OF MIR-122 FROM EXTRACELLULAR VESICLES AND SERUM FROM EXPERIMENTAL MODELS OF MASLD

The microRNAs were extracted from EVs using the miRNeasy serum/plasma kit (Qiagen, USA). The cel-miR-39 (1.6×10^8 copies) spike-in control (Qiagen, USA) was added as an internal reference for normalization of technical variations between samples, following the manufacturer's instructions. The cDNA conversion was performed from 10 ng of total RNA using TaqMan microRNA reverse transcription kits (Applied Biosystems, USA). The gene expression analysis of miR-122 and its normalizer cel-miR-39 (assay ID: 002245 and 000200 respectively), was performed using qRT-PCR with TaqMan probes (Applied Biosystems, USA). The values will be calculated using the $2^{-(\Delta\Delta Ct)}$ formula.

EXCLI Journal 2025;24:1438-1455 – ISSN 1611-2156
Received: July 02, 2025, accepted: October 07, 2025, published: October 23, 2025

RESULT

Supplementary Table 2a: Demographic variables, comorbidities, and liver function scores across MASLD stages

# Variables		Steatosis (n=50) n (%)	MASH (n=49) n (%)	Cirrhosis (n=50) n (%)	HCC (n=20) n (%)	*p*
Gender	Female	34 (20.2 %)	31 (18.5 %)	33 (19.6 %)	10 (6.0 %)	0.543
	Male	16 (9.5 %)	17 (10.1 %)	17 (10.1 %)	10 (6.0 %)	
Active Smoker	Smoker	8 (5.1 %)	2 (1.3 %)	4 (2.5 %)	0 (0 %)	0.467
	Non-smoker	27 (17.2 %)	31 (19.7 %)	32 (20.4 %)	6 (3.8 %)	
	ex-smoker	15 (9.6 %)	15 (9.6 %)	14 (8.9 %)	3 (1.9 %)	
Alcohol consumption	Yes	3 (1.9 %)	4 (2.5 %)	3 (1.9 %)	1 (0.6 %)	0.666
	No	44 (28 %)	41 (26.1 %)	44 (28 %)	6 (3.8 %)	
	Former alcoholic	3 (1.9 %)	3 (1.9 %)	3 (1.9 %)	2 (1.3 %)	
Hypertension	Hypertensive	33 (19.6 %)	38 (22.6 %)	43 (25.6 %)	18 (10.7 %)	**0.048***
	Non-hypertensive	17 (10.1 %)	10 (6 %)	7 (4.2 %)	2 (1.2 %)	
Diabetes	Diabetic	28 (16.7 %)	30 (17.9 %)	38 (22.6 %)	17 (10.1 %)	**0.047***
	Non-diabetic	22 (13.1 %)	18 (10.7 %)	12 (7.1 %)	3 (1.8 %)	
MetS	Yes	50 (29.8 %)	48 (28.6 %)	48 (28.6 %)	19 (11.3 %)	0.225
	No	0 (0 %)	0 (0 %)	2 (1.2 %)	1 (0.6 %)	
HBV positive (only anti- HBcIgG positive)	Yes	4 (2.4 %)	1 (0.6 %)	1 (0.6 %)	0 (0 %)	0.235
	No	46 (27.4 %)	47 (28 %)	49 (29.2 %)	20 (11.9 %)	
Anti-HCV positive (PCR negative)	Yes	7 (4.2 %)	4 (2.4 %)	1 (0.6 %)	0 (0 %)	0.666
	No	43 (25.6 %)	44 (26.2 %)	49 (29.2 %)	20 (11.9 %)	
MELD Score		-	-	49 (98.0 %)	20 (100 %)	**0.015***
Child-Pugh classification		-	-	50 (100 %)	9 (45 %)	**0.017***

HBV: hepatitis B virus; HCC: hepatocellular carcinoma; HCV: hepatitis C virus; MASLD: metabolic-dysfunction-associated steatotic liver disease; MASH: metabolic dysfunction-associated steatohepatitis; MetS: metabolic syndrome. * $p < 0.05$, ** $p < 0.001$, Chi-square tests, Kruskal–Walli's test for MELD and Child-Pugh scores; post-hoc Mann–Whitney U test for pairwise comparison between cirrhosis and HCC (Child-Pugh). "–" indicates that the variable was not applicable to the respective group.

Supplementary Table 2b: Logistic regression analysis of comorbidities associated with different stages of MASLD

# Variables	Groups	Odds Ratio (IC 95 %)	*p*
Hypertension	Steatosis	4.636 (1.056, 20.341)	0.056
	MASH	3.400 (0.518, 10.840)	0.296
	Cirrhosis	1.465 (0.260, 8.269)	0.653
	HCC	1.789 (0.458, 6.980)	0.411
Diabetes	Steatosis	4.452 (1.030, 19.327)	**0.030***
	MASH	3.400 (0.694, 16.681)	0.078
	Cirrhosis	1.789 (0.458, 6.980)	0.411
	HCC	1.789 (0.458, 6.980)	0.411

HCC: hepatocellular carcinoma; MASH: metabolic dysfunction-associated steatohepatitis. * $p < 0.05$, ** $p < 0.001$; Chi-square test and logistic regression were used.

EXCLI Journal 2025;24:1438-1455 – ISSN 1611-2156
Received: July 02, 2025, accepted: October 07, 2025, published: October 23, 2025

Supplementary Table 2c: Biochemical variables assessed at different stages of MASLD

# Variables	Steatosis (n=50)	MASH (n=49)	Cirrhosis (n=50)	HCC (n=20)	*p*
	Median (IQR)	Median (IQR)	Median (IQR)	Median (IQR)	
ALT (U/L)	32.50 (33.81 - 49.05) [a]	35.00 (34.99 - 51.53) [a]	31.00 (27.57 - 55.42) [a]	35.00 (20.09 - 54.47) [a]	0,517
AST (U/L)	24.00 (19.40 - 66.50) [a]	31.00 (30.44 - 39.59) [a,b]	37.00 (30.04 - 68.66) [b]	43.00 (22.89 - 67.96) [b]	**0,001****
GGT (U/L)	62.00 (54.10 - 203.83) [a]	54.50 (53,77 - 106.23) [a]	114.50 (121.78 - 242.50) [b]	264.00 (2.74 - 606.68) [b]	**0,000****
ALP (U/L)	87.00 (81.86 - 101.74) [a,c]	82.50 (81.24 - 100.88) [a]	97.00 (105.22 - 150.69) [b,c]	181.00 (81.06 - 334.64) [b]	**0,000****
Albumin (g/L)	4.40 (4.29 - 4.50) [a]	4.30 (4.22 - 4.42) [a]	4.00 (3.67 - 4.09) [b]	3.80 (3.06 - 4.41) [b]	**0,000****
Creatinine (mg/dL)	0.76 (0.70 - 1.19) [a]	0.82 (0.79 - 0.90) [a,b]	0.89 (0.85 - 1.16) [a,b]	1.11 (0.40 - 3.28) [b]	**0,003***
Total Bilirubin (mg/dL)	0.50 (0.46 - 0.66) [a]	0.50 (0.47 - 0.60) [a,b]	0.75 (0.76 - 1.04) [b]	1.10 (0.21 - 2.72) [b]	**0,000****
Indirect Bilirubin (mg/dL)	0.30 (0.29 - 0.43) [a]	0.35 (0.28 - 0.53) [a]	0.50 (0.45 - 0.63) [b]	0.50 (0.20 - 1.16) [a,b]	**0,003***
Triglycerides (mg/dL)	132.50 (131.37 - 195.49) [a,b]	155.00 (149.59 - 185.58) [a]	121.00 (114.01 - 154.61) [b]	92.00 (72.92 - 147.64) [b]	**0,002***
TC (mg/dL)	181.00 (169.37 - 192.71) [a]	180.00 (170.13 - 196.64) [a]	153.50 (151.38 - 177.90) [b]	145.00 (118.54 - 188.02) [b]	**0,048***
HDL (mg/dL)	47.00 (42.11 - 48.49) [a]	40.50 (38.84 - 44.33) [a]	37.50 (39.15 - 48.63) [a]	31.00 (24.10 - 40.46) [a]	0,225
LDL (mg/dL)	102.50 (91.16 - 112.03) [a]	109.10 (98.14 - 122.65) [a]	89.80 (85.57 - 107.18) [a]	91.00 (69.66 - 129.35) [a]	0,323
Glucose (mg/dL)	119.50 (120.61- 154.47) [a]	117.50 (114.82 - 142.17) [a]	129.50 (129.87 - 174.37) [a]	135.00 (76.80 - 268.33) [a]	0,528
White Blood Cell Count	6660.00 (4791.59 - 7054.70) [a]	7110.00 (5113.14 - 7217.25) [a]	5.38 (-20.04 - 775.29) [b]	6.56 (3.40 - 14.19) [b]	**0,000****
Platelet Count	241.00 (213.92 - 258.59) [a]	235.00 (222.65 - 261.73) [a]	113.50 (102.29 - 135.61) [b]	159.00 (86.14 - 361.85) [a]	**0,000****

ALP: alkaline phosphatase; ALT: alanine aminotransferase; AST: aspartate aminotransferase; GGT: gamma-glutamyl transferase; HCC: hepatocellular carcinoma; HDL: high-density lipoprotein; LDL: low-density lipoprotein; MASH: metabolic dysfunction-associated steatohepatitis; TC: total cholesterol. *a,b* Different letters indicate statistically significant differences. * $p < 0.05$, ** $p < 0.001$; Kruskal–Wallis test.

EXCLI Journal 2025;24:1438-1455 – ISSN 1611-2156
Received: July 02, 2025, accepted: October 07, 2025, published: October 23, 2025

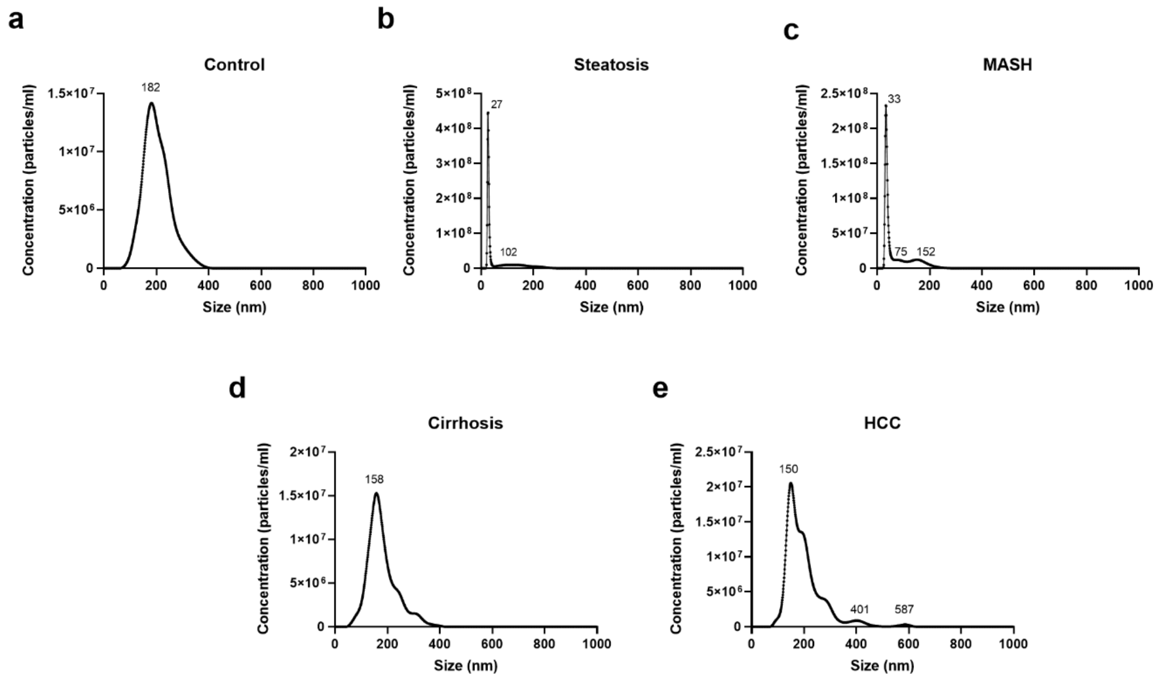

Figure S1: Size distribution curves representative of vesicular populations in each patient group. (**a**) Control group. (**b**) Steatotic group. (**c**) MASH group. (**d**) Cirrhosis group. (**e**) HCC group. Size values <150 nm correspond to exosomes, and those in the range of 150-1000 nm correspond to microvesicles. HCC: hepatocellular carcinoma; MASH: metabolic dysfunction associated steatohepatitis.

Received: July 02, 2025, accepted: October 07, 2025, published: October 23, 2025

FIGURES S2, S3, AND S4. UNEDITED FULL GELS FOR FIGURE 2D – WESTERN BLOTTING

Each figure shows the name of the protein expressed in each gel, along with the bands for each group. The first five bands in each figure correspond to Figure 2d of the manuscript. For analyses, protein loading was normalized to 40 µg of protein. A total of n=9 samples were used, with two samples per experimental group except for the hepatocellular carcinoma group. A separate membrane was prepared for each antibody.

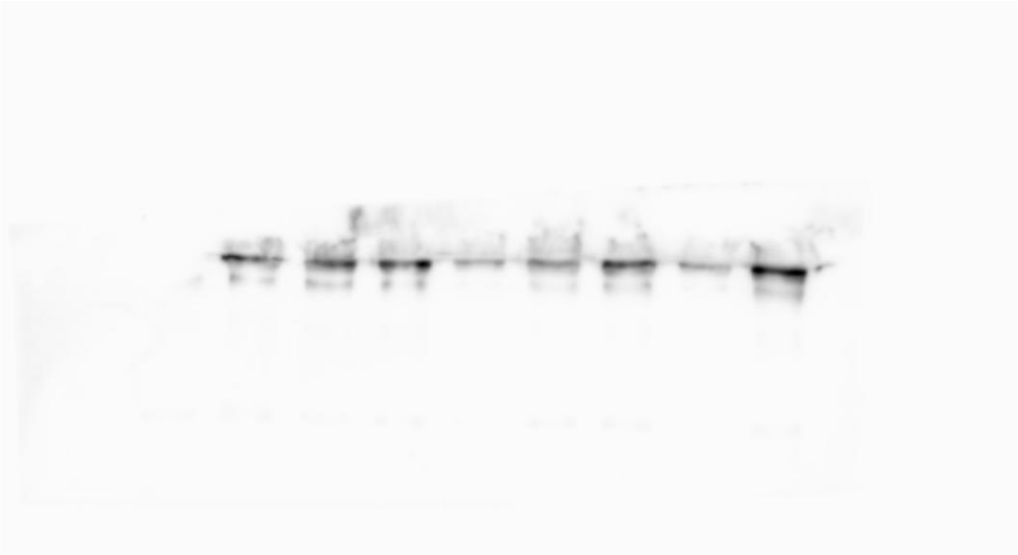

Figure S2: GM130 Protein Expression (~130 kDa). Nine lanes are shown corresponding to the following samples: 1. Control; 2. Steatosis; 3. MASH; 4. Cirrhosis; 5. HCC; 6. Control; 7. Steatosis; 8. MASH; 9. Cirrhosis.

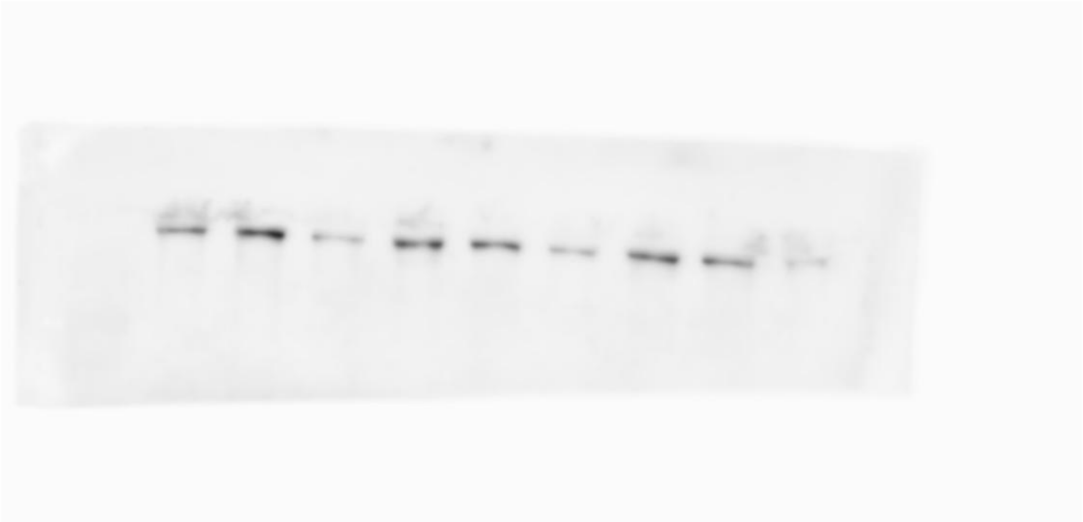

Figure S3: Alix Protein Expression (~95 kDa). Nine lanes are shown corresponding to the following samples: 1. Control; 2. Steatosis; 3. MASH; 4. Cirrhosis; 5. HCC; 6. Control; 7. Steatosis; 8. MASH; 9. Cirrhosis.

EXCLI Journal 2025;24:1438-1455 – ISSN 1611-2156
Received: July 02, 2025, accepted: October 07, 2025, published: October 23, 2025

Figure S4: Annexin Protein Expression (~35 kDa). Nine lanes are shown corresponding to the following samples: 1. Control; 2. Steatosis; 3. MASH; 4. Cirrhosis; 5. HCC; 6. Control; 7. Steatosis; 8. MASH; 9. Cirrhosis.

Experimental model miR-122 expression in serum and EVs

Our results showed that miR-122 was expressed within EVs; however, there was no significant difference between the groups (Supplementary Figure 5a). When we evaluated the serum expression of mir-122, we observed a significant increase in the MASLD groups compared to their respective controls, and the MASLD-28 group showed a significant increase compared to MASLD-16 ($p<0.001$) (Supplementary Figure 5b). Given this difference in expression between EVs and serum, we proceeded to compare the expression of miR-122 between these two sample types only in the diseased groups. Therefore, within the MASLD-28 group, there was a significant increase in miR-122 expression in the serum [(10.472 ± 6.579), $p<0.05$] compared to within EVs (1.272 ± 0.639). However, there were no differences at week 16 (Supplementary Figure 5c).

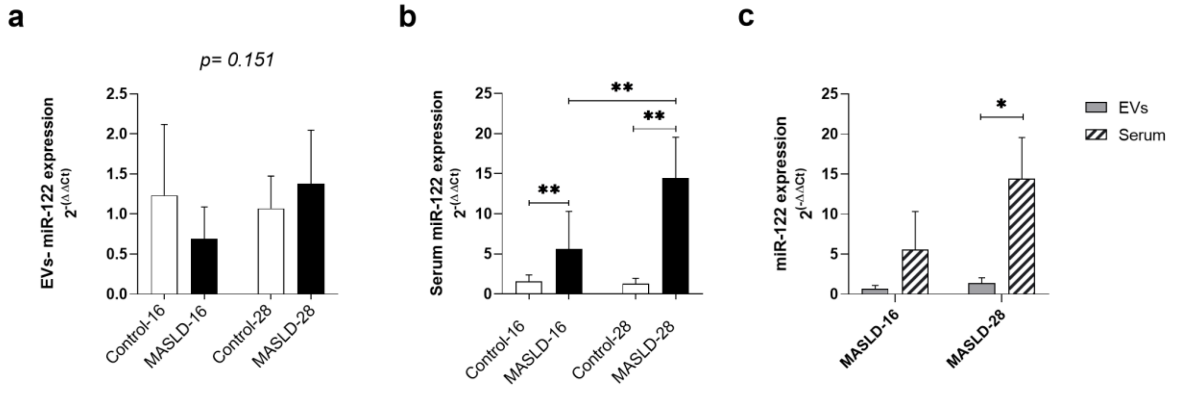

Figure S5: Expression of miR-122 in experimental MASLD models. (**a**) miR-122 within EVs. (**b**) miR-122 in serum. (**c**) Comparison of miR-122 expression in EVs and serum in MASLD groups. Data were presented as mean ± standard deviation using the Two-Way ANOVA Test with Tukey's Post-hoc Test and Paired-samples T test. Statistical significance of ** $p \leq 0.001$, * $p \leq 0.05$. EVs: extracellular vesicles; MASLD: metabolic-dysfunction-associated steatotic liver disease

Supplementary Table 3: Correlation Analysis Between Extracellular Vesicles, microRNAs, and Biochemical/Clinical Variables in MASLD

·Variables[a]	concentration (particles/ml)	size EVs (nm)	miR4758-EVs	miR122-EVs	miR4758-serum	miR122-serum	miR188-serum	miR-1226-serum
ALT (U/L)	-0,007	**-,206***	-0,048	0,030	0,256	0,188	0,189	-0,002
AST (U/L)	-0,045	-0,009	0,022	0,004	0,096	-0,249	0,307	-0,238
GGT (U/L)	-0,104	0,028	0,072	0,431	0,170	0,056	-0,320	-0,378
ALP (U/L)	-0,077	0,076	0,216	0,399	0,193	-0,035	0,186	-0,179
Albumin (g/L)	0,192	**-,317****	-0,162	0,249	-0,158	0,262	-0,171	0,208
Creatinine (mg/dL)	0,085	0,071	-0,135	**-,538***	-0,110	-0,217	0,164	-0,278
Total Bilirubin (mg/dL)	0,062	0,190	**,423***	0,044	0,301	-0,263	0,191	-0,153
Triglycerides (mg/dL)	0,036	**-,345****	-0,136	0,318	-0,255	-0,006	-0,266	0,251
TC (mg/dL)	0,018	-0,096	-0,125	0,305	0,197	0,392	-0,003	0,269
HDL (mg/dL)	0,041	0,131	-0,029	0,080	-0,016	0,402	0,319	0,253
LDL (mg/dL)	-0,026	0,008	-0,102	0,262	0,217	0,096	-0,111	-0,103
Glucose (mg/dL)	0,015	-0,071	**,392***	-0,192	0,012	-0,241	-0,040	-0,135
MELD Score	0,285	-0,099	0,434	-	0,494	0,095	0,522	0,277
Child-Pugh classification	0,173	0,085	0,438	-	0,279	-0,079	0,283	-0,079

[a]Variables were represented for Spearman’s correlation coefficient, moderate (0.3< r <0.6), strong (0.6< r <0.9), or very strong (0.9< r <1.0). *Correlation is significant at the 0.05 level. **Correlation is significant at the 0.01 level. Correlations were identified in bold; positive correlations were additionally highlighted in light gray, and negative correlations in dark gray. Correlations with MELD and Child-Pugh scores were not calculated for all variables, as these scores are not applicable to all MASLD patients.

ALP: alkaline phosphatase, ALT: alanine aminotransferase, AST: aspartate aminotransferase, GGT: gamma-glutamyl transferase, HCC: hepatocellular carcinoma, HDL: high-density lipoprotein, LDL: low-density lipoprotein, TC: total cholesterol

EXCLI Journal 2025;24:1438-1455 – ISSN 1611-2156
Received: July 02, 2025, accepted: October 07, 2025, published: October 23, 2025

Supplementary Table 3a: Correlation between EVs, microRNAs, and biochemical variables in Steatosis group

Variables[a]	concentration (particles/ml)	size EVs (nm)	miR4758-EVs	miR122-EVs	miR4758-serum	miR122-serum	miR188-serum	miR-1226-serum
ALT (U/L)	-0,155	**-,471***	-0,164	0,043	-0,486	0,429	0,086	-0,771
AST (U/L)	-0,179	-0,148	0,176	-0,067	-0,543	-0,086	0,600	-0,257
GGT (U/L)	-0,170	**-,443***	-0,167	0,183	-0,300	0,300	-0,600	-0,600
ALP (U/L)	-0,229	0,050	0,182	0,085	-0,771	-0,143	0,657	-0,714
Albumin (g/L)	0,110	-0,391	-0,520	0,440	0,232	-0,348	-0,319	-0,087
Creatinine (mg/dL)	0,172	0,059	0,345	-0,382	-0,371	0,257	0,200	-0,086
Total Bilirubin (mg/dL)	0,040	0,072	**,706***	0,149	0,120	-0,598	0,000	0,598
Triglycerides (mg/dL)	-0,011	-0,324	-0,267	0,333	0,714	-0,371	-0,543	0,543
TC (mg/dL)	-0,096	**-,462***	-0,430	0,103	0,257	-0,086	0,086	-0,086
HDL (mg/dL)	0,041	-0,044	-0,347	-0,286	-0,086	0,029	**,886***	-0,029
LDL (mg/dL)	-0,084	-,434*	-0,224	0,236	-0,029	0,543	-0,257	-0,543
Glucose (mg/dL)	0,168	-0,179	0,292	-0,134	0,371	0,543	-0,086	0,314

[a]Variables were represented for Spearman's correlation coefficient, moderate (0.3< r <0.6), strong (0.6< r <0.9), or very strong (0.9< r <1.0). *Correlation is significant at the 0.05 level. **Correlation is significant at the 0.01 level. Correlations were identified in bold; positive correlations were additionally highlighted in light gray, and negative correlations in dark gray. MELD and Child-Pugh scores were not evaluated in this group, as they are not applicable.

ALP: alkaline phosphatase, ALT: alanine aminotransferase, AST: aspartate aminotransferase, GGT: gamma-glutamyl transferase, HCC: hepatocellular carcinoma, HDL: high-density lipoprotein, LDL: low-density lipoprotein, TC: total cholesterol

EXCLI Journal 2025;24:1438-1455 – ISSN 1611-2156
Received: July 02, 2025, accepted: October 07, 2025, published: October 23, 2025

Supplementary Table 3b: Correlation between EVs, microRNAs, and biochemical variables in MASH group

Variables	concentration (particles/ml)	size EVs (nm)	miR4758-EVs	miR122-EVs	miR4758-serum	miR122-serum	miR188-serum	miR-1226-serum
ALT (U/L)	-0,033	-0,040	-0,333	0,158	0,200	-0,429	0,200	0,500
AST (U/L)	0,100	-0,100	-0,249	0,162	0,100	-0,543	0,100	1,000**
GGT (U/L)	-0,254	0,107	-0,030	0,365	-0,300	-0,600	-0,500	-1,000**
ALP (U/L)	-0,336	0,037	0,170	0,555	-0,200	**-,886***	-0,800	-1,000**
Albumin (g/L)	0,241	-0,113	-0,195	0,092	0,289	**-,828***	-0,289	
Creatinine (mg/dL)	0,252	0,018	-0,345	-0,565	0,700	0,657	1,000**	0,500
Total Bilirubin (mg/dL)	0,365	0,015	-0,131	-0,122	-0,289	0,098	0,000	0,866
Triglycerides (mg/dL)	0,068	-0,140	0,079	0,243	-0,700	-0,257	-0,700	-0,500
TC (mg/dL)	-0,115	0,364	-0,224	0,267	-0,200	0,200	-0,200	-0,500
HDL (mg/dL)	-0,206	0,336	0,049	-0,052	-0,100	0,486	0,100	0,500
LDL (mg/dL)	-0,124	0,330	-0,139	0,182	-0,100	0,257	0,100	0,500
Glucose (mg/dL)	0,119	-0,206	0,333	-0,237	-0,600	0,486	-0,100	-0,500

[a]Variables were represented for Spearman's correlation coefficient, moderate (0.3< r <0.6), strong (0.6< r <0.9), or very strong (0.9< r <1.0). *Correlation is significant at the 0.05 level. **Correlation is significant at the 0.01 level. Correlations were identified in bold; positive correlations were additionally highlighted in light gray, and negative correlations in dark gray. MELD and Child-Pugh scores were not evaluated in this group, as they are not applicable. ρ = 1.000 values based on less than five observations are not statistically reliable and were excluded from interpretation.

ALP: alkaline phosphatase, ALT: alanine aminotransferase, AST: aspartate aminotransferase, GGT: gamma-glutamyl transferase, HCC: hepatocellular carcinoma, HDL: high-density lipoprotein, LDL: low-density lipoprotein, TC: total cholesterol

EXCLI Journal 2025;24:1438-1455 – ISSN 1611-2156
Received: July 02, 2025, accepted: October 07, 2025, published: October 23, 2025

Supplementary Table 3c: Correlation between EVs, microRNAs, and biochemical variables in Cirrhosis group

Variables[a]	concentration (particles/ml)	size EVs (nm)	miR4758-EVs	miR4758-serum	miR122-serum	miR188-serum	miR-1226-serum
ALT (U/L)	0,083	-0,286	0,268	0,232	0,377	0,406	0,200
AST (U/L)	0,137	-0,217	0,300	0,314	0,314	0,314	0,200
GGT (U/L)	0,267	-0,324	0,117	0,314	0,771	-0,200	0,400
ALP (U/L)	0,125	-0,036	-0,151	0,371	0,371	0,714	1,000**
Albumin (g/L)	0,216	-0,247	-0,092	0,232	0,232	-0,029	-0,200
Creatinine (mg/dL)	-0,100	-0,092	-0,183	-0,143	0,314	-0,143	0,000
Total Bilirubin (mg/dL)	0,100	-0,236	0,525	0,696	0,087	0,000	-0,105
Triglycerides (mg/dL)	0,231	-0,362	-0,524	-0,700	0,300	0,200	0,500
TC (mg/dL)	0,028	0,081	0,092	-0,058	0,725	0,377	1,000**
HDL (mg/dL)	-0,165	**,457***	-0,133	-0,429	-0,143	-0,257	-0,400
LDL (mg/dL)	0,038	0,030	-0,008	0,116	0,116	0,203	0,800
Glucose (mg/dL)	0,131	**-,516****	**,667***	**,829***	0,200	-0,371	0,000
MELD Score	0,144	-0,056	0,434	0,667	0,522	-0,029	0,400
Child-Pugh classification	0,021	0,163	0,438	0,525	0,309	-0,216	-0,258

[a]Variables were represented for Spearman's correlation coefficient, moderate (0.3< r <0.6), strong (0.6< r <0.9), or very strong (0.9< r <1.0). *Correlation is significant at the 0.05 level. **Correlation is significant at the 0.01 level. Correlations were identified in bold; positive correlations were additionally highlighted in light gray, and negative correlations in dark gray. ρ = 1.000 values based on less than five observations are not statistically reliable and were excluded from interpretation.

ALP: alkaline phosphatase, ALT: alanine aminotransferase, AST: aspartate aminotransferase, GGT: gamma-glutamyl transferase, HCC: hepatocellular carcinoma, HDL: high-density lipoprotein, LDL: low-density lipoprotein, TC: total cholesterol

EXCLI Journal 2025;24:1438-1455 – ISSN 1611-2156
Received: July 02, 2025, accepted: October 07, 2025, published: October 23, 2025

Supplementary Table 3d: Correlation between EVs, microRNAs, and biochemical variables in HCC group

Variables[a]	concentration (particles/ml)	size EVs (nm)	miR4758-serum	miR122-serum	miR188-serum	miR-1226-serum
ALT (U/L)	0,036	0,002	0,486	0,086	-0,086	0,429
AST (U/L)	0,145	-0,188	0,371	0,086	0,314	0,314
GGT (U/L)	-0,005	0,125	0,464	0,754	0,493	0,580
ALP (U/L)	-0,167	0,290	0,486	0,486	0,257	0,657
Albumin (g/L)	-0,106	0,057	-0,638	-0,348	-0,725	-0,638
Creatinine (mg/dL)	0,180	-0,214	-0,143	-0,600	0,257	-0,086
Total Bilirubin (mg/dL)	-0,169	0,263	0,464	-0,116	0,319	0,116
Triglycerides (mg/dL)	0,320	-0,166	-0,410	-0,154	-0,359	-0,821
TC (mg/dL)	0,106	-0,056	0,300	**,900***	-0,200	0,300
HDL (mg/dL)	-0,025	0,047	0,400	-0,500	0,600	0,500
LDL (mg/dL)	0,043	-0,094	0,300	**,900***	-0,200	0,300
Glucose (mg/dL)	-0,203	0,143	-0,314	-0,429	-0,371	-0,371
MELD Score	0,190	-0,131	0,522	-0,058	0,493	0,290
Child-Pugh classification	0,113	0,151	0,247	-0,278	0,339	0,123

[a]Variables were represented for Spearman's correlation coefficient, moderate (0.3< r <0.6), strong (0.6< r <0.9), or very strong (0.9< r <1.0). *Correlation is significant at the 0.05 level. **Correlation is significant at the 0.01 level. Correlations were identified in bold; positive correlations were additionally highlighted in light gray, and negative correlations in dark gray. Correlations involving miR-4758–EVs and miR-122–EVs were not calculated in the HCC group, as miR-4758–EVs was not expressed in this group and miR-122–EVs was not detected.

ALP: alkaline phosphatase, ALT: alanine aminotransferase, AST: aspartate aminotransferase, GGT: gamma-glutamyl transferase, HCC: hepatocellular carcinoma, HDL: high-density lipoprotein, LDL: low-density lipoprotein, TC: total cholesterol.

EXCLI Journal 2025;24:1438-1455 – ISSN 1611-2156
Received: July 02, 2025, accepted: October 07, 2025, published: October 23, 2025

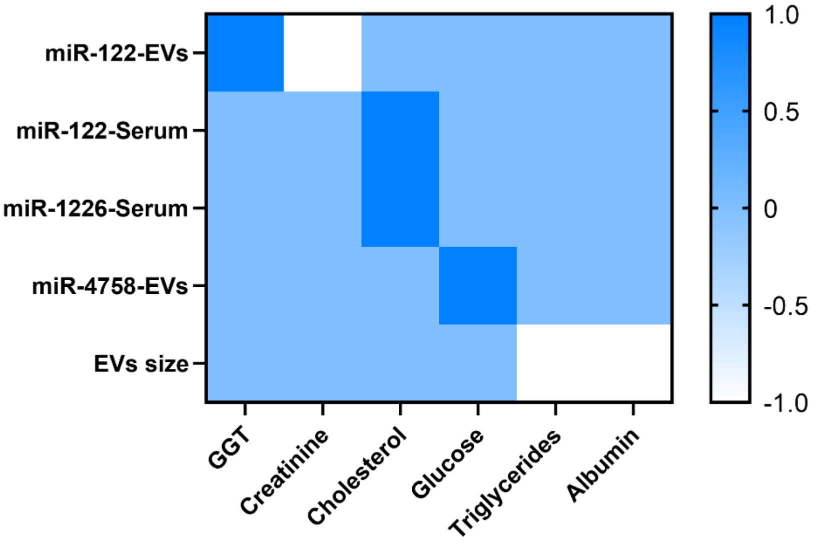

Figure S6: Differential EV and miRNA profiles by high vs. low biochemical marker levels. Each cell represents the direction of statistically significant differences between patients with high versus low levels of each biochemical marker. +1 (blue): higher in high marker group; –1 (white): lower in high marker group; 0 (light blue): not significant. Mann–Whitney U test. Statistical significance: $p \leq 0.05$. EVs: extracellular vesicles; GGT: gamma-glutamyl transferase.

Design interacting networks for each selected microRNA

For miR188 and miR-1226, one PPI network each was predicted, while two PPI networks were predicted for miR-4758 (Supplementary Figure 7a). Subsequently, centrality analysis was conducted, identifying 29 key targets for miR188, 18 for miR-1226, and 16 for miR-4758 (Supplementary Figure 7b). Out of the key targets identified for each microRNA, only 8 for miR-4758, 5 for miR-1226, and 8 for miR-188 have been tested and described in the literature regarding MASLD (Supplementary Table 4).

EXCLI Journal 2025;24:1438-1455 – ISSN 1611-2156
Received: July 02, 2025, accepted: October 07, 2025, published: October 23, 2025

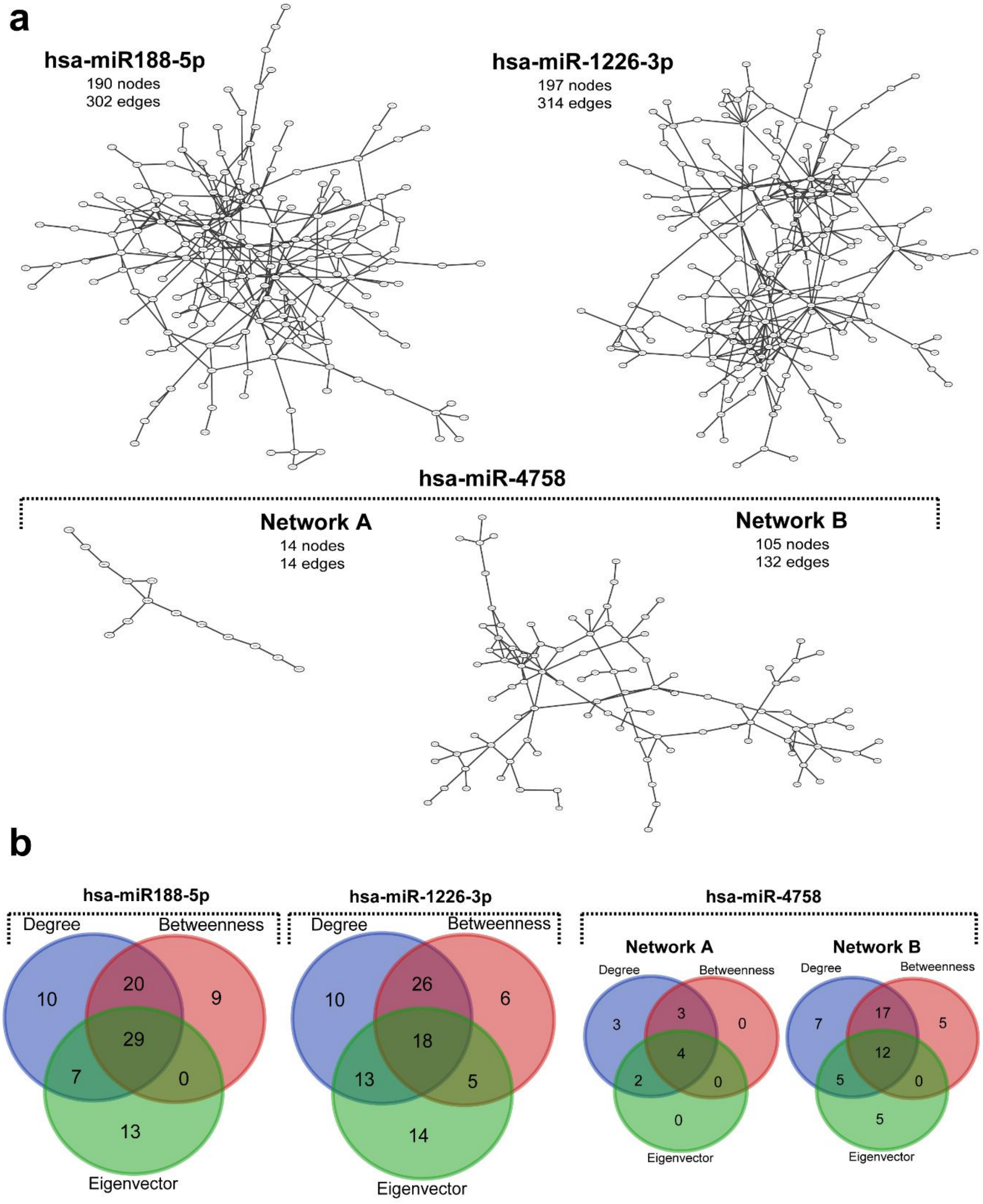

Figure S7: Systems biology analysis identifying highly connected predicted target genes. (**a**) Protein-Protein Interaction (PPI) networks were obtained by combining MiRWalk target predictions and STRINGApp for each selected microRNA. (**b**) Venn diagrams were used to predict nodes that are more relevant based on degree, betweenness, and eigenvector parameters for each PPI network from microRNA targets

Supplementary Table 4: microRNA targets and their associated genes in MASLD

microRNAs	Symbol	Name of Gene	Stage of the disease	Description
**miR-4758 (8)**	ADORA2A	Adenosine A2a receptor (A2AR)	Cirrhosis and HCC	Encodes for the Adenosine A2a receptor (A2AR), which has anti-inflammatory and anti-obesogenic functions. Low expression of ADORA2A is associated with the development of cirrhosis, hepatic inflammation, and HCC (Allard et al., 2023)
	CEBPB	CCAAT Enhancer Binding Protein Beta	MASH	Transcription factor CCAAT/enhancer-binding protein (C/EBP), abundant in the liver and related to the infiltration of immune cells and maintenance of liver function, may promote MASLD through inflammatory activation of the liver and lipid metabolism (Han et al., 2022; Zhao et al., 2019)
	IRF1	Interferon regulatory factor 1	MASH-HCC	IRF1, a key member of the Interferon Regulatory Transcription Factor (IFR) protein family, is involved in the regulation of hepatic inflammation in NASH and in the proapoptotic and anti-proliferative response of HCC tumor cells (Yan et al., 2021; Zhang et al., 2022)
	CDC25C	Cell Division Cycle 25C	HCC	Regulator protein of the cell cycle and is closely related to the size, differentiation, and grades of tumors in HCC (Liu et al., 2020; Xun et al., 2020)
	FADD	Fas associated via death domain	HCC	Adaptor protein involved in the process of programmed cell death related to the development of HCC (Wei et al., 2023)

	IL15	Interleukin 15	MASH (inflammation and lipids)	Cytokine is involved in lipid accumulation, inflammatory response, and immune cell recruitment, promoting MASLD (Cepero-Donates et al., 2016).
	TIMP3	TIMP Metallopeptidase Inhibitor 3	Steatosis, MASH	Acts as a key regulator in the liver during obesity, preventing excessive lipid accumulation and the development of HCC (Casagrande et al., 2017).
	MCHR1	Melanin concentrating hormone receptor 1	Steatosis, MASH	Cyclic neuropeptide with a role in controlling eating behavior, energy homeostasis, hepatic lipid metabolism, and inflammation. The presence of MCHR1 is associated with the development of MASLD (Kawata et al., 2017).
**miR-1226 (5)**	ADRB3	Adrenoceptor beta 3	Steatosis, MASH	The ADRB3 gene has polymorphisms that are implicated in the onset of obesity and insulin resistance, factors associated with an increased risk of MASLD (Sakamoto et al., 2019).
	VCAM1	Vascular cell adhesion molecule 1	MASH	VCAM1 is found in the sinusoidal endothelial cells of the liver, and its increased expression is associated with triggering an inflammatory immune response, hepatic damage, and fibrosis (Furuta et al., 2021).
	GNAS	GNAS complex locus	HCC	The GNAS gene encodes the alpha subunit of the stimulatory G protein (Gsα) and mediates the proliferation and invasion of hepatocellular carcinoma cell lines induced by inflammation (Ding et al., 2020).
	CXCR2	C-X-C motif chemokine receptor 2	MASH-HCC	A crucial receptor for neutrophil recruitment during acute injuries and is associated with NASH-HCC. Increased CXCR2 expression may lead to a protumoral state (Leslie et al., 2022).
	RUNX2	RUNX Family Transcription Factor 2	HCC	Implicated in promoting migration and invasion of HCC cells. This gene is associated with mechanisms that degrade extracellular matrix components and promote metastasis in HCC cells (Wang et al., 2016).

**miR-188 (8)**	GPX7	Glutathione Peroxidase 7	MASH-Fibrosis	Antioxidant enzyme and its overexpression in hepatic cells have demonstrated an anti-fibrotic effect, reducing the production of inflammatory cytokines and inhibiting ROS (Kim et al., 2020).
	PRKCD	Protein Kinase C Delta	HCC	Its high expression is implicated in the migration and invasion of tumor hepatic cells (Qin et al., 2021).
	eEF1A1	Eukaryotic Translation Elongation Factor 1 Alpha 1	HCC	eEF1A1 overexpression has been correlated with a worse prognosis in patients with HCC (Chen et al., 2018).
	FBXW7	F-box And WD Repeat Domain Containing 7	Steatosis	It is involved in glucose and lipid homeostasis during Metabolic Syndrome (MS), playing a protective role in hepatic steatosis by reducing inflammation and insulin resistance (Zhang et al., 2019).
	ACAA2	Acetyl-CoA Acyltransferase 2	Steatosis	Participates in the oxidation metabolism of fatty acids in the liver; therefore, its decrease is associated with the progression of hepatic steatosis and promotes HCC progression (Wu et al., 2023).
	NOTCH4	Notch Receptor 4	MASH-Fibrosis	Plays a crucial role in the Notch pathway. Activation of this pathway is associated with the loss of functional liver identity and the promotion of inflammation and liver fibrosis related to NASH (Zhu et al., 2021).
	PARP1	Poly(ADP-Ribose) Polymerase 1	Steatosis	It is a cellular stress sensor that can be activated by oxidative, metabolic, and genotoxic stresses. Its activation promotes lipid accumulation and liver inflammation, worsening hepatic steatosis (Huang et al., 2017).
	AKR1B1	Aldo-Keto Reductase Family 1, Member B1	Steatosis	The presence of AKR1B1 is associated with metabolic reprogramming in MASLD, promoting lipid accumulation in hepatic cells and contributing to the development and progression of hepatic steatosis (Syamprasad et al., 2024)

REFERENCES

Allard B, Jacoberger-Foissac C, Cousineau I, Bareche Y, Buisseret L, Chrobak P, et al. Adenosine A2A receptor is a tumor suppressor of NASH-associated hepatocellular carcinoma. Cell Rep Med. 2023;4(9): 101188. doi: 10.1016/j.xcrm.2023.101188.

Casagrande V, Mauriello A, Bischetti S, Mavilio M, Federici M, Menghini R. Hepatocyte-specific TIMP3 expression prevents diet-dependent fatty liver disease and hepatocellular carcinoma. Sci Rep. 2017;7(1):799. doi: 10.1038/s41598-017-06439-x.

Cepero-Donates Y, Lacraz G, Ghobadi F, Rakotoarivelo V, Orkhis S, Mayhue M, et al. Interleukin-15-mediated inflammation promotes non-alcoholic fatty liver disease. Cytokine. 2016;82:102–111. doi: 10.1016/j.cyto.2016.01.020.

Chen SL, Lu SX, Liu LL, Wang CH, Yang X, Zhang ZY, et al. eEF1A1 overexpression enhances tumor progression and indicates poor prognosis in hepatocellular carcinoma. Transl Oncol. 2018;11(1):125–131. doi: 10.1016/j.tranon.2017.11.001.

de Freitas LBR, Longo L, Filippi-Chiela E, de Souza VEG, Behrens L, Pereira MHM, et al. Ornithine aspartate and vitamin E combination has beneficial effects on cardiovascular risk factors in an animal model of nonalcoholic fatty liver disease in rats. Biomolecules. 2022;12(12):1773. doi: 10.3390/biom12121773.

Ding H, Zhang X, Su Y, Jia C, Dai C. GNAS promotes inflammation-related hepatocellular carcinoma progression by promoting STAT3 activation. Cell Mol Biol Lett. 2020;25(1):44. doi:10.1186/s11658-020-00204-1.

Furuta K, Guo Q, Pavelko KD, Lee JH, Robertson KD, Nakao Y, et al. Lipid-induced endothelial vascular cell adhesion molecule 1 promotes nonalcoholic steatohepatitis pathogenesis. J Clin Invest. 2021; 131(6): e143690. doi: 10.1172/JCI143690.

Han N, He J, Shi L, Zhang M, Zheng J, Fan Y. Identification of biomarkers in nonalcoholic fatty liver disease: a machine learning method and experimental study. Front Genet. 2022;13:1020899. doi: 10.3389/fgene.2022.1020899.

Huang K, Du M, Tan X, Yang L, Li X, Jiang Y, et al. PARP1-mediated PPARα poly(ADP-ribosyl)ation suppresses fatty acid oxidation in non-alcoholic fatty liver disease. J Hepatol. 2017;66(5):962–977. doi: 10.1016/j.jhep.2016.11.020.

Jordan F, Sharma A, Scardoni G, Tosadori G, Faizan M, Spoto F, et al. Biological network analysis with CentiScaPe: centralities and experimental dataset integration. F1000Res. 2015;4:480. doi: 10.12688/f1000research.4477.1.

Kawata Y, Okuda S, Hotta N, Igawa H, Takahashi M, Ikoma M, et al. A novel and selective melanin-concentrating hormone receptor 1 antagonist ameliorates obesity and hepatic steatosis in diet-induced obese rodent models. Eur J Pharmacol. 2017;796:45–53. doi: 10.1016/j.ejphar.2016.12.018.

Keingeski MB, Longo L, Brum da Silva Nunes V, Figueiró F, Dallemole DR, Pohlmann AR, et al. Extracellular vesicles and their correlation with inflammatory factors in an experimental model of steatotic liver disease associated with metabolic dysfunction. Metab Syndr Relat Disord. 2024;22 (5):394-401. doi: 10.1089/met.2023.0284.

Kim HJ, Lee Y, Fang S, Kim W, Kim HJ, Kim JW. GPx7 ameliorates non-alcoholic steatohepatitis by regulating oxidative stress. BMB Rep. 2020;53(6): 317–322. doi: 10.5483/BMBRep.2020.53.6.280.

Leslie J, Mackey JBG, Jamieson T, Ramon-Gil E, Drake TM, Fercoq F, et al. CXCR2 inhibition enables NASH-HCC immunotherapy. Gut. 2022;71(10): 2093–2106. doi: 10.1136/gutjnl-2021-326259.

Liu K, Zheng M, Lu R, Du J, Zhao Q, Li Z, et al. The role of CDC25C in cell cycle regulation and clinical cancer therapy: a systematic review. Cancer Cell Int. 2020;20(1):242. doi: 10.1186/s12935-020-01304-w.

Longo L, Ferrari JT, Rampelotto PH, Dellavia GH, Pasqualotto A, Oliveira CP, et al. Gut dysbiosis and increased intestinal permeability drive microRNAs, NLRP3 inflammasome and liver fibrosis in a nutritional model of non-alcoholic steatohepatitis in adult male rats. Clin Exp Gastroenterol. 2020;13:351–368. doi: 10.2147/CEG.S262879.

Qin F, Zhang J, Gong J, Zhang W. Identification and validation of a prognostic model based on three autophagy-related genes in hepatocellular carcinoma. Biomed Res Int. 2021;2021:5564040. doi: 10.1155/2021/5564040.

Sakamoto Y, Oniki K, Kumagae N, Morita K, Otake K, Ogata Y, et al. Beta-3-adrenergic receptor rs4994 polymorphism is a potential biomarker for the development of nonalcoholic fatty liver disease in overweight/obese individuals. Dis Markers. 2019;2019: 4065327. doi: 10.1155/2019/4065327.

Scardoni G, Lau C. Centralities based analysis of complex networks. In: New Frontiers in Graph Theory. Rijeka: InTech, 2012. doi: 10.5772/35846.

Scardoni G, Tosadori G, Faizan M, Spoto F, Fabbri F, Laudanna C. Biological network analysis with CentiScaPe: centralities and experimental dataset integration. F1000Res. 2014;3:139. doi: 10.12688/f1000research.4477.1.

Syamprasad NP, Jain S, Rajdev B, Panda SR, Kumar GJ, Shaik KM, et al. AKR1B1 drives hyperglycemia-induced metabolic reprogramming in MASLD-associated hepatocellular carcinoma. JHEP Rep. 2024; 6(2):100974. doi: 10.1016/j.jhepr.2023.100974.

Théry C, Witwer KW, Aikawa E, Alcaraz MJ, Anderson JD, Andriantsitohaina R, et al. Minimal information for studies of extracellular vesicles 2018 (MISEV2018): a position statement of the International Society for Extracellular Vesicles and update of the MISEV2014 guidelines. J Extracell Vesicles. 2018;7(1):1535750. doi: 10.1080/20013078.2018.1535750.

Wang Q, Yu W, Huang T, Zhu Y, Huang C. RUNX2 promotes hepatocellular carcinoma cell migration and invasion by upregulating MMP9 expression. Oncol Rep. 2016;36(5):2777–2784. doi: 10.3892/or.2016.5101.

Wei Y, Lan C, Yang C, Liao X, Zhou X, Huang X, et al. Robust analysis of a novel PANoptosis-related prognostic gene signature model for hepatocellular carcinoma immune infiltration and therapeutic response. Sci Rep. 2023;13(1):41670. doi: 10.1038/s41598-023-41670-9.

Wu D, Liao G, Yao Y, Huang L, Dong B, Ma Y, et al. Downregulated acetyl-CoA acyltransferase 2 promoted the progression of hepatocellular carcinoma and participated in the formation of immunosuppressive microenvironment. J Hepatocell Carcinoma. 2023;10: 1327–1339. doi: 10.2147/JHC.S418429.

Xun R, Lu H, Wang X. Identification of CDC25C as a potential biomarker in hepatocellular carcinoma using bioinformatics analysis. Technol Cancer Res Treat. 2020;19:1533033820967474. doi: 10.1177/1533033820967474.

Yan Y, Zheng L, Du Q, Yazdani H, Dong K, Guo Y, et al. Interferon regulatory factor 1 activates anti-tumor immunity via CXCL10/CXCR3 axis in hepatocellular carcinoma. Cancer Lett. 2021;506:95–106. doi: 10.1016/j.canlet.2021.03.002.

Zhang C, Chen F, Feng L, Shan Q, Zheng GH, Wang YJ, et al. FBXW7 suppresses HMGB1-mediated innate immune signaling to attenuate hepatic inflammation and insulin resistance in a mouse model of nonalcoholic fatty liver disease. Mol Med (Cambridge, Mass). 2019;25(1):99. doi: 10.1186/s10020-019-0099-9.

Zhang C, Liu S, Yang M. The role of interferon regulatory factors in non-alcoholic fatty liver disease and non-alcoholic steatohepatitis. Gastroenterol Insights. 2022;13(2):148–161. doi: 10.3390/gastroent13020016.

Zhao X, Reebye V, Hitchen P, Fan J, Jiang H, Sætrom P, et al. Mechanisms involved in the activation of C/EBPα by small activating RNA in hepatocellular carcinoma. Oncogene. 2019;38(18):3446–3457. doi: 10.1038/s41388-018-0665-6.

Zhu C, Ho YJ, Salomao MA, Dapito DH, Bartolome A, Schwabe RF, et al. Notch activity characterizes a common hepatocellular carcinoma subtype with unique molecular and clinicopathologic features. J Hepatol. 2021;74(3):613–626. doi: 10.1016/j.jhep.2020.09.032.
